# Evaluation of guidelines on antimicrobials use in food-producing animals: a systematic review

**DOI:** 10.1186/s42522-025-00160-w

**Published:** 2025-08-14

**Authors:** Jacinta Oliveira Pinho, Ana Isabel Plácido, Alexandra Monteiro, Rafaela Nogueira, Paula Alexandra Oliveira, Ana Claúdia Coelho, Adolfo Figueiras, Fátima Roque, Maria Teresa Herdeiro

**Affiliations:** 1 Department of Medical Sciences, Institute of Biomedicine (iBiMED), University of Aveiro, Campus Universitário de Santiago, Agra do Crasto, Aveiro, 3810-193 Portugal; 2 BRIDGES - Biotechnology Research, Innovation and Design for Health Products, Polytechnic University of Guarda, Avenida Dr. Francisco Sá Carneiro, N. 50, Guarda, 6300-559 Portugal; 3 Centre for the Research and Technology of Agro-Environmental and Biological Sciences (CITAB), University of Trás-Os-Montes and Alto Douro (UTAD), Vila Real, Portugal; 4 Animal and Veterinary Research Centre (CECAV), Department of Veterinary Sciences, University of Trás-Os-Montes and Alto Douro (UTAD), Vila Real, Portugal; 5 Department of Veterinary Sciences, School of Agrarian and Veterinary Sciences, University of Trás-os-Montes and Alto Douro, Vila Real, Portugal; 6 Associate Laboratory for Animal and Veterinary Sciences (AL4AnimalS), Faculty of Veterinary Medicine, University of Lisboa, Lisboa, Portugal; 7 Department of Preventive Medicine and Public Health, University of Santiago de Compostela; Health Research Institute of Santiago de Compostela (IDIS), Santiago de Compostela, Spain; 8 Consortium for Biomedical Research in Epidemiology and Public Health (CIBER en Epidemiología y Salud Pública-CIBERESP), Carlos III Health Institute, Madrid, Spain

**Keywords:** Antimicrobial resistance, One Health approach, Veterinary guidelines, Food-producing animals, OECD-related countries

## Abstract

**Background:**

Antimicrobial resistance (AMR) is a global public health problem due to misuse/overuse of antimicrobials. The interplay between humans, animals, and the environment requires a One Health approach for effective AMR control. We focused this research on antimicrobial use in food-producing animals (bovine, caprine, equine, ovine, and swine) to assess the compliance of Organisation for Economic Co-operation and Development (OECD) countries (members, partners, and candidates) with international guidelines: Codex Alimentarius: Code of Practice to Minimize and Contain Foodborne Antimicrobial Resistance, and the Terrestrial Animal Health Code.

**Methods:**

For this systematic review (PROSPERO CRD42024535461), between February 1 and June 30 of 2024, guidelines were searched on: governmental websites associated with health and veterinary sectors, veterinary organizations specified by the government or included in the country’s National Action Plan for AMR, and the global repository of available guidelines for responsible use of antimicrobials in animal health. Three researchers performed data extraction and AGREE II appraisal was conducted by two researchers.

**Results:**

Of the 49 OECD countries, 37 presented guidelines (*n* = 82) for responsible antimicrobial use in the analyzed species, with bovine and swine being the most represented. The highest number of published guidelines was observed between 2017–2020. The number of clinical and non-clinical guidelines were 43 and 37, respectively, emphasizing the need for veterinarian-directed recommendations.

**Conclusions:**

The AMR challenge, the interdependence of countries, and the trade of animal-derived products should encourage national initiatives to develop and implement guidelines for the judicious use of antimicrobials in animal production. Due to OECD countries’ disparities in terms of culture, internal policies, attitudes and perceptions about AMR, and financial resources, this process needs to be gradual and tailored for each case. Therefore, communication and collaboration between countries and stakeholders are essential.

**Supplementary Information:**

The online version contains supplementary material available at 10.1186/s42522-025-00160-w.

## Introduction

Antimicrobials are essential in human and animal health care, encompassing antibiotic, antifungal, antiparasitic, and antiviral drugs [1]. Despite being a lifesaving breakthrough, the misuse and overuse of antimicrobials has led to antimicrobial resistance (AMR), placed in the top ten worldwide public health concerns by the World Health Organization (WHO) [2]. Resistant microorganisms can naturally arise in humans, animals, food, plants, and the environment [3], and this has also been reported during clinical treatments with antimicrobials [4]. However, AMR development is aggravated by the excessive and inadequate use of antimicrobials in human medicine, veterinary, and agriculture practices [3].

In this context, a One Health approach [5] is required to address AMR, being defined by WHO as “an integrated, unifying approach to balance and optimize the health of people, animals and ecosystems” [5]. Antimicrobial stewardship initiatives have emerged to tackle AMR within the One Health framework and are implemented at multiple levels, including national, regional, community, and institutional settings [6]. The development and effectiveness of these initiatives depend on national policies, regulatory authorities, human healthcare workers, veterinarians, farmers, industry actors, and the general public [6].

Among these efforts, national and international guidelines serve as critical starting points for countries to build internal structures and shape their antimicrobial use (AMU) governance. While these documents alone do not constitute a fully operational response to AMR, they offer an essential foundation by outlining key principles, minimum standards, and strategic direction. Establishing such guidance is particularly important for countries where institutional capacity, policy coherence, or awareness on AMR remains limited [7]. Expanding antimicrobial stewardship frameworks on a global scale, especially in low-middle income countries (LMICs), requires not only human and financial resources, but also international guidance and frameworks [8]. Cooperation, coordinated action, and strategic planning of international organizations [5, 9] enable the design and implementation of clinical guidelines to standardize procedures, to support clinical decisions of health professionals, and to promote more efficient and effective practices [10]. In veterinary medicine, the Food and Agriculture Organization (FAO), WHO, and the World Organisation for Animal Health (WOAH) have published documents providing guidance for the responsible use of antimicrobials – the Codex Alimentarius: Code of Practice to Minimize and Contain Foodborne Antimicrobial Resistance [11] (FAO/WHO) and the Terrestrial Animal Health Code [12] (WOAH).

These international veterinary guidelines gather updated literature, incorporating new discoveries and advances to improve healthcare quality and safety [10]. Each country is responsible for adapting these international recommendations to its own legislation and epidemiological status, updating them as appropriate, and effectively disseminating the information across health professionals and/or stakeholders for a successful application [13].

To our knowledge, there is a lack of studies broadly analyzing the existence and agreement/disagreement of national guidelines with those established by FAO, WHO, and WOAH. To address this gap, our major goal was to assess guidelines of Organisation for Economic Co-operation and Development (OECD) countries for veterinary care in bovine, caprine, equine, ovine, and swine. We also aimed to: (i) summarize key characteristics of guidelines; (ii) assess the quality of clinical and non-clinical guidelines; and (iii) analyze the compliance of clinical guidelines with international recommendations.

## Materials and methods

### Search strategy

We conducted a systematic review in accordance with Preferred Reporting Items for Systematic Reviews and Meta-Analyses (PRISMA) statement [14], registered in the PROSPERO database (CRD42024535461) [15]. It focused on guidelines for the prescription of antimicrobials in food-producing animals (bovine, caprine, equine, ovine, and swine). The search was limited to countries that are OECD members, accession candidates, and key partners (*n* = 49 countries; appendix pp 10-11), and it was performed between February 1 and June 30 of 2024.

The search strategy for identifying potential guidelines involved three key approaches: first, searching the websites of relevant government ministries responsible for medical and veterinary sectors. Second, a search was conducted in veterinary organizations indicated by the government or included in the National Action Plans (NAPs) of Antimicrobial Resistance published in the WHO Library of NAPs. Third, the global repository of available guidelines for responsible use of antimicrobials in animal health, created by the World Veterinary Association (WVA) in collaboration with the WOAH [16], was consulted. The search on the theme was performed in the respective countries’ original languages. The PICO question used as the basis of this work was: What guidelines have OECD countries adopted for antimicrobial stewardship in food-producing animals? This question is divided as follows: P (population): All stakeholders involved in developing, revising, and implementing guidelines for the responsible and prudent use of antimicrobials in food-producing animals; I (intervention): Prescription of antimicrobials in veterinary, specifically in food-producing animals; C (comparator): international recommendations on antimicrobial use, as established by WHO, WOAH, and FAO; O (outcome): existence/non-existence of guidelines, their specificities, and agreement/disagreement with international recommendations.

### Eligibility criteria

#### Inclusion criteria

(i) Guidelines and stewardship documents with a focus on the use of antimicrobials in food-producing animals (bovine, swine, equine, ovine, and caprine) concerning OECD-related countries (members, partners, and candidates); (ii) Documents published on the websites of the government and the ministries/agencies responsible for regulating the veterinary sector and/or pharmaceutical sector concerning OECD-related countries; (iii) Documents published by government-indicated veterinary associations and/or organizations of OECD-related countries and/or included in the NAPs on antimicrobial resistance published on the WHO Library of NAPs.

#### Exclusion criteria

(i) Guidelines and stewardship documents addressing other medicines or medicines in general in food-producing animals; (ii) Guidelines and stewardship documents with a focus on the use of antimicrobials in other animals; (iii) Document specific to a procedure/treatment/active substance/disease; (iv) Documents published on websites other than by the official government and the ministries/agencies responsible for regulating the veterinary sector and/or pharmaceutical sector; (v) Documents published by veterinary associations and/or organizations that are not indicated by the governments and/or included in the NAPs; (vi) Documents that are not antimicrobial stewardship guidelines (e.g.: regulatory documents, reports, NAPs, scientific communication, notification/notice/advisory document, policies, guidance on food processing practices for farmers, articles).

A preliminary screening by title and aim was conducted, followed by a thorough evaluation of the full-text version to determine if the guidelines met the inclusion criteria. Three authors, RN, AM, and JOP, performed independent assessments, with a fourth researcher consulted in cases of disagreement. If multiple versions of a guideline were available, only the most recent was considered (retrieved guidelines were published between 2000 and 2024). All documents not meeting the eligibility criteria are listed in the appendix (pp 4-5), with the reasons for exclusion. Following the completion of the searches, countries without appropriate guidelines for responsible antimicrobial use in the species under investigation were also documented. After accessing and documenting the available guidelines, these were reviewed, either in their original language (English, Portuguese, and Spanish) or after being translated to English through an online translation engine (one author used Google Translate and the others used DeepL for comparison) [17]. The usefulness and suitability of both tools have been confirmed by several researchers [18–20].

### Data analysis

Data extraction was performed independently by three researchers, RN, AM, and JOP, using a data collection template (Appendix 2, p 3). The following data was extracted from the documents: country, year of publication, title, author(s), target species, target audience, scope, focus (antimicrobials or antibiotics), and source (government or other). OECD status (member, candidate, and/or key partner) was also described. Pertinent details were also recorded: the existence or not of medically important antimicrobials (MIAs) classification in the guidelines, and the listing of their active ingredients.

The instrument Appraisal of Guidelines for Research & Evaluation (AGREE) II [21] was used to assess the quality of retrieved documents. AGREE II is constituted by 23 appraisal criteria (items) divided into six domains, each of which “captures a distinctive aspect of guideline quality”: Domain 1, scope and purpose; Domain 2, stakeholder involvement; Domain 3, rigor of development; Domain 4, clarity of presentation; Domain 5, applicability; Domain 6, editorial independence. The items in each domain are assessed on a 7-point Likert scale (1- strongly disagree to 7- strongly agree) [22]. Final domain scores were calculated by combining individual item scores in a domain from a blind analysis of two appraisers and expressing them as a percentage of the maximum score for that domain [21].

Next, RN, AM, and JOP reviewed the clinical guidelines for agreement/disagreement with 10 listed recommendations from each international source – Codex Alimentarius: Code of Practice to Minimize and Contain Foodborne Antimicrobial Resistance, and the Terrestrial Animal Health Code, by FAO/WHO and WOAH.

## Results

### Search results

**Fig. 1:**
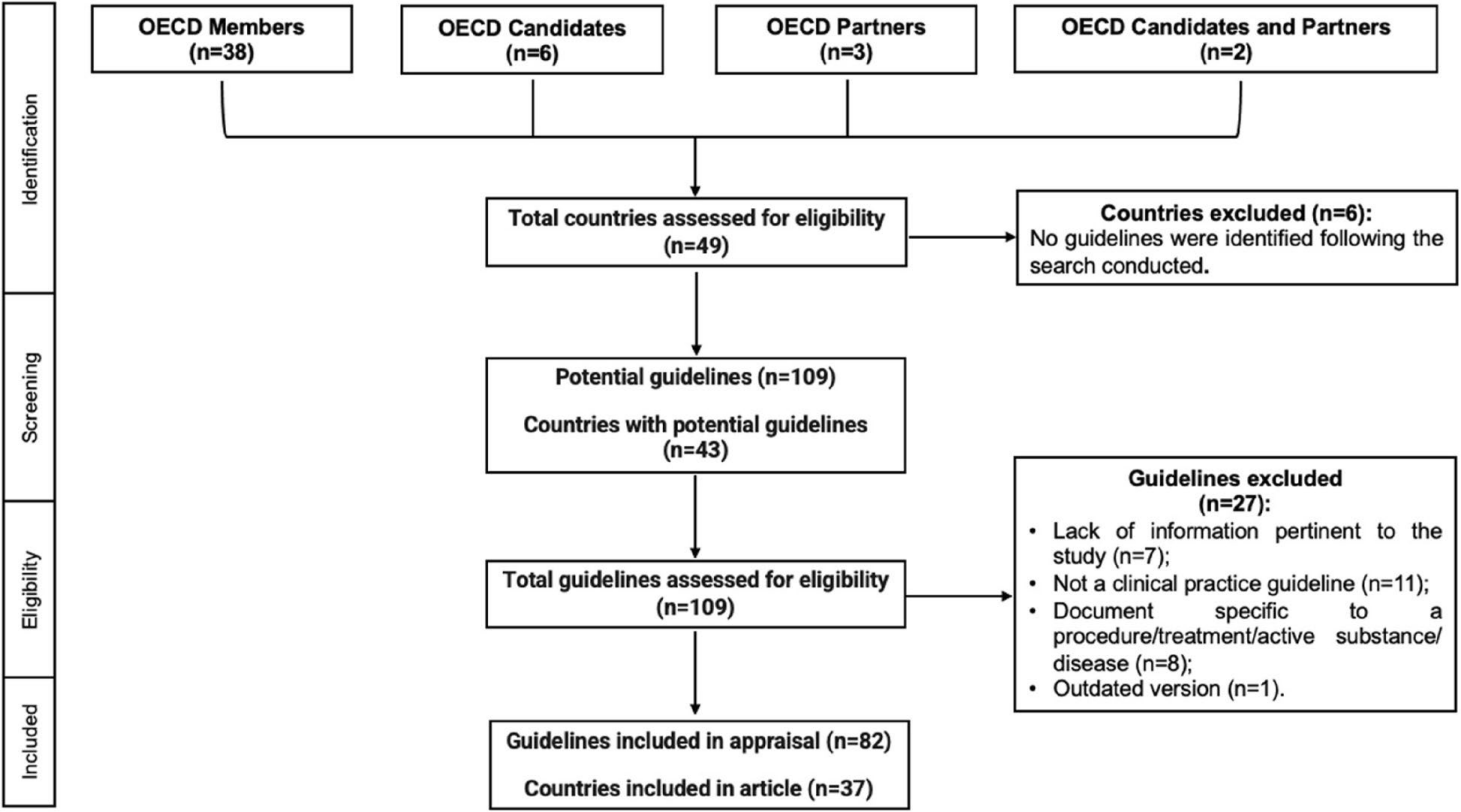
Flow diagram of guideline selection

The search in governmental and non-governmental sources of the 49 OECD countries resulted in the exclusion of six countries, for which no documents related to antimicrobial use in defined food-producing animals were found. The remaining 43 OECD countries yielded a total of 109 documents (Fig. 1). After full-text screening, 82 (75·23%) were eligible as guidelines, belonging to 37 countries (out of 43): 31 members, 1 key partner (South Africa), 3 candidates (Bulgaria, Romania, Thailand), and 2 candidates/key partners (Brazil and Indonesia). The complete list of guidelines is in the appendix (pp 6-8). Out of the 49 OECD countries, a total of 12 did not present antimicrobial stewardship guidelines for the animal species under evaluation (appendix p 9). Moreover, all OECD countries have developed NAPs (except Israel, which NAP is under development [23]; appendix pp 10-11). The content analysis of NAPs was not performed since it was out of this work’s scope.

### Main characteristics of retrieved guidelines

**Fig. 2:**
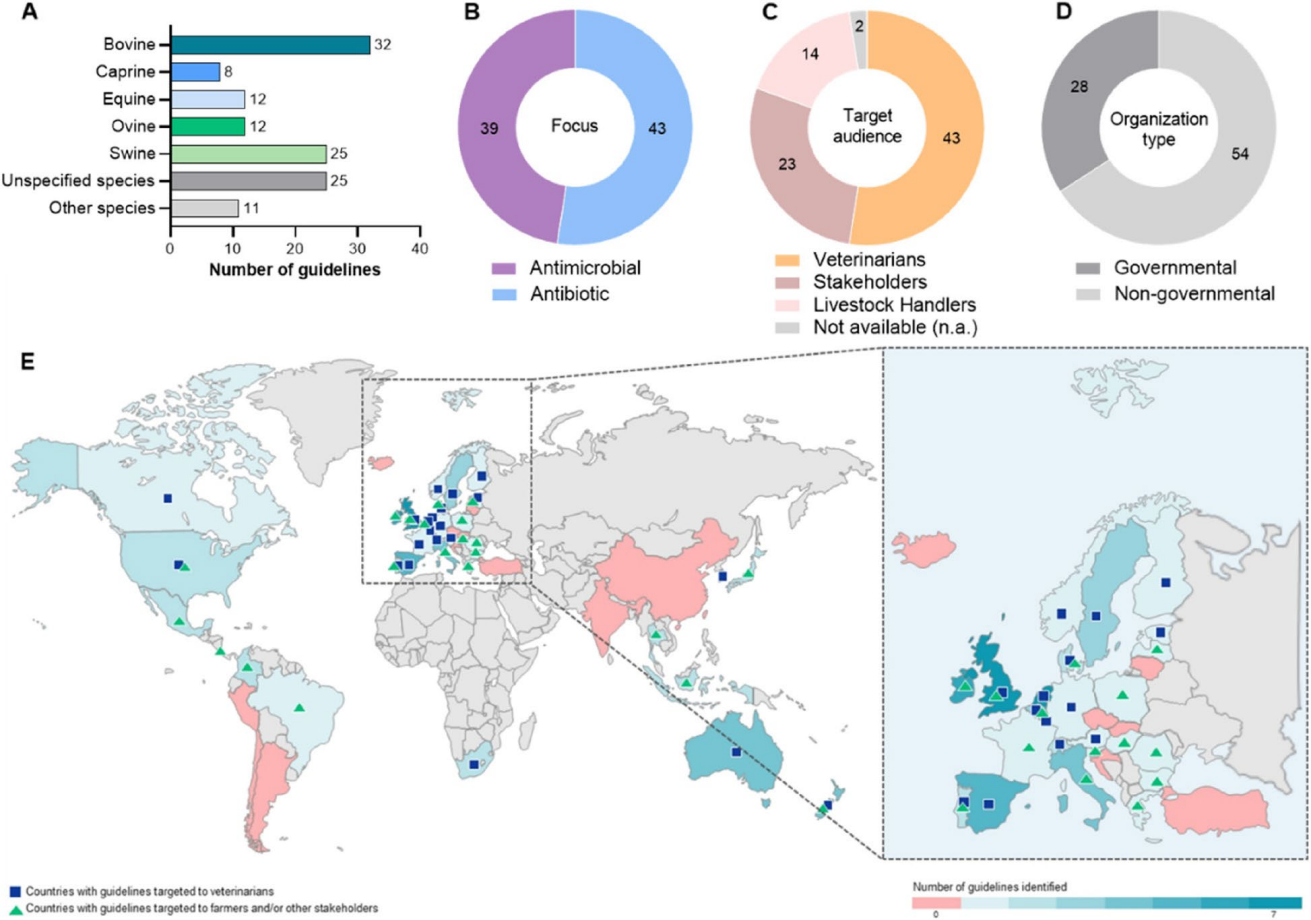
Major characteristics of the 82 included guidelines and corresponding world map. **A** Target species, **B** Focus, **C** Target audience, **D** Type of organization that elaborated and published the guideline, and (**E**) World map of guidelines’ distribution and target audience. **A** Some guidelines would include, in the same document, the 5 main species: bovine, caprine, equine, ovine, and swine. In these cases, each species was accounted for individually within the same document; Other species: other livestock animals (different than the main 5 addressed) and pets, including poultry, dogs, cats, fish, rabbits, chinchillas, hamsters, deer, fur animals, bees, small rodents, and New World camelids; Livestock handlers: owners, farmers, producers, animal keepers, breeders, etc.; Stakeholders: veterinarians, livestock handlers, pharmaceutical industries, health managers, regulatory authorities, laboratories, etc.

Table 1 and Fig. 2 outline the main characteristics of the 82 guidelines. Of these, 39 (47·56%) were dedicated to the prudent use of antimicrobials, while 43 (52·44%) focused on the use of antibiotics. Of the 82 included guidelines, 25 (31·71%) were non-species specific. The remaining ones addressed the specific species, either individually or within the same document, resulting in a higher number of guidelines *per* species (*n* = 125; Table 1 and Fig. 2A): bovine (*n* = 32; [24–55]), caprine (*n* = 8; [34, 35, 40, 45, 50, 52, 56, 57]), equine (*n* = 12; [26, 34, 35, 40, 46, 58–63]), ovine (*n* = 12; [26, 35, 40, 45, 50, 52, 53, 57, 64–67]), and/or swine (*n* = 25; [26, 28, 29, 33–35, 40, 45, 46, 50–52, 68–80]) (Fig. 2A). A total of 13 guidelines would include in the same document these different species (Table 1) [26, 28, 29, 33–35, 40, 45, 46, 50–53]. Other countries, in turn, provided separate guidelines for each species (Table 1), namely Australia, Belgium, Colombia, Costa Rica, Denmark, Ireland, Italy, Korea, Mexico, Netherlands, New Zealand, Portugal, Spain, and the UK [24, 25, 27, 30–32, 36–39, 41–43, 47–49, 54–76, 78–80]. Throughout 10 documents, other species were also mentioned (Table 1), namely poultry [26, 28, 29, 33–35, 40, 46], fish [28, 33–35], dogs [26, 34, 35, 40, 46], cats [26, 34, 35, 40, 46], rabbits [26, 46], deer [34, 44], bees [34], hamsters [26], chinchillas [26], fur animals [34], small rodents [46], and New World camelids. [52] More than half of guidelines specifically addressed veterinarians (*n* = 43), while 37 were non-clinical, being directed towards stakeholders (*n* = 23) and livestock handlers (*n* = 14) (Fig. 2B). The stakeholders include veterinarians, farmers, livestock handlers, regulatory authorities, pharmaceutical industry, laboratories, distributors and sellers, pharmacists, veterinarian and producer associations, academia, and general public (Fig. 2B). The remaining two guidelines did not specify the target audience, i.e., veterinarians, livestock handlers, or stakeholders. In total, 28 guidelines were retrieved from governmental sources, while the other 54 were from non-governmental entities (Fig. 2C).

**Table 1:** Main characteristics of the included guidelines on the prudent use of antimicrobials in food-producing animals

Country	Last Update	Target Audience	Target Species	Antimicrobial (AM)/Antibiotic (AB)	Organization	Ref
Australia	2020	Veterinarians	Sw	AM	Ngov	[68]
	2024	Veterinarians	Ov	AM	Ngov	[64]
	2024	Veterinarians	Bov	AM	Ngov	[24]
	2024	Veterinarians	Bov	AM	Ngov	[25]
Austria	2024	Veterinarians	Bov, Sw, Ov, Eq, other (rabbit, hamster, chinchillas, poultry, dogs, cats)	AB	Gov	[26]
Belgium	2021	Stakeholders	Bov	AB	Ngov	[27]
	2021	Stakeholders	Sw	AB	Ngov	[69]
	2021	Veterinarians	Eq	AB	Ngov	[58]
Brazil^a^	2022	Stakeholders	Bov, Sw, other (poultry, fish)	AB	Gov	[28]
Bulgaria^b^	2021	Stakeholders	Unspecified species	AM	Gov	[81]
Canada	2008	Veterinarians	Bov, Sw, other (poultry)	AM	Ngov	[29]
Colombia	2019	Stakeholders	Sw	AM	Ngov	[70]
	2020	Livestock handlers	Bov	AB	Ngov	[30]
Costa Rica	2018	Livestock handlers	Bov	AM	Ngov	[31]
Denmark	2013	Stakeholders	Bov	AB	Ngov	[32]
	2018	Veterinarians	Sw	AM	Gov	[71]
Estonia	2020	Veterinarians	Bov, Sw, other (poultry, fish)	AB	Ngov	[33]
Finland	2018	Veterinarians	Bov, Sw, Cap, Eq, other (poultry, fish, bees, dogs, cats, fur animals, deer)	AM	Gov	[34]
France	2009	Stakeholders	Unspecified species	AB	Ngov	[82]
Germany	2015	Veterinarians	Bov, Sw, Ov, Cap, Eq, other (poultry, fish, dogs, cats)	AM	Ngov	[35]
Greece	2020	n.a	Unspecified species	AM	Gov	[83]
Hungary	2020	Veterinarians	Unspecified species	AB	Gov	[84]
Indonesia^a^	2017	Stakeholders	Unspecified species	AM	Ngov	[97]^d^
	2021	Stakeholders	Unspecified species	AB	Gov	[98]
Ireland	2018	Livestock handlers	Unspecified species	AB	Ngov	[92]
	2019	Livestock handlers	Sw	AM	Ngov	[72]
	2019	Livestock handlers	Bov	AM	Ngov	[36]
	2020	Livestock handlers	Bov	AM	Ngov	[37]
	2020	Livestock handlers	Ov	AM	Ngov	[65]
	2022	Livestock handlers	Eq	AM	Gov	[59]
Italy	2018	Stakeholders	Unspecified species	AB	Ngov	[85]
	2018	Stakeholders	Unspecified species	AB	Gov	[86]
	2022	Stakeholders	Sw	AM	Ngov	[73]
	2023	Stakeholders	Bov	AB	Gov	[38]
Japan	2000	Stakeholders	Unspecified species	AB	Gov	[90]
	2013	Stakeholders	Unspecified species	AB	Gov	[91]
Korea (Republic of Korea)	2020	Veterinarians	Bov	AB	Gov	[39]
	2020	Veterinarians	Sw	AB	Gov	[74]
	2022	Veterinarians	Bov, Sw, Eq, Ov, Cap, other (poultry, dogs, cats)	AB	Gov	[40]
Latvia	2018	Stakeholders	Unspecified species	AM	Gov	[87]
Luxembourg	2022	Veterinarians	Unspecified species	AB	Gov	[93]
Mexico	2021	Stakeholders	Unspecified species	AM	Gov	[94]
	2023	Stakeholders	Sw	AM	Gov	[75]
Netherlands	2015	Veterinarians	Unspecified species	AM	Ngov	[119]
	2019	Veterinarians	Sw	AB	Ngov	[76]
	2019	Veterinarians	Cap	AB	Ngov	[56]
	2019	Veterinarians	Bov	AB	Ngov	[41]
	2019	Veterinarians	Ov	AB	Ngov	[66]
	2021	Veterinarians	Eq	AB	Ngov	[60]
	2023	Veterinarians	Bov	AB	Ngov	[42]
New Zealand	2018	Veterinarians	Bov	AB	Ngov	[43]
	2018	Stakeholders	Bov, other (deer)	AM	Ngov	[44]
	2018	Veterinarians	Eq	AB	Ngov	[61]
	2020	Veterinarians	Sw	AM	Ngov	[77]
Norway	2022	Veterinarians	Bov, Sw, Ov, Cap	AB	Ngov	[45]
Poland	2020	Veterinarians	Bov, Sw, Eq, other (poultry, rabbit, dogs, cats, small rodents)	AM	Ngov	[46]
Portugal	2021	Stakeholders	Unspecified species	AM	Gov	[96]
	2023	Veterinarians	Bov	AM	Gov	[47]
Romania^b^	2020	Stakeholders	Unspecified species	AM	Gov	[88]
Slovenia	n.a	Stakeholders	Unspecified species	AB	Gov	[89]
South Africa^c^	2002	Veterinarians	Unspecified species	AM	Ngov	[99]
	2016	Veterinarians	Sw	AM	Ngov	[78]
Spain	2017	Veterinarians	Bov	AB	Ngov	[48]
	2017	Veterinarians	Eq	AB	Ngov	[62]
	2017	Veterinarians	Sw	AB	Ngov	[79]
	2021	Veterinarians	Bov	AM	Gov	[49]
	2021	Veterinarians	Ov, Cap	AB	Ngov	[57]
Sweden	2013	Veterinarians	Eq	AB	Ngov	[63]
	2017	Veterinarians	Bov, Sw, Ov, Cap	AB	Ngov	[50]
	2019	Veterinarians	Bov, Sw	AB	Ngov	[51]
Switzerland	2022	Veterinarians	Bov, Sw, Ov, Cap, other (New World camelids)	AB	Ngov	[52]
	2022	Veterinarians	Unspecified species	AB	Ngov	[100]
Thailand^b^	2009	Stakeholders	Unspecified species	AM	Gov	[101]
	2017	Stakeholders	Unspecified species	AM	Ngov	[97]^d^
United Kingdom (UK)	2014	Livestock handlers	Unspecified species	AB	Ngov	[102]
	2018	Livestock handlers	Sw	AB	Ngov	[80]
	2019	Veterinarians	Unspecified species	AM	Ngov	[103]
	2019	Livestock handlers	Ov	AB	Ngov	[67]
	2020	Livestock handlers	Bov, Ov	AB	Ngov	[53]
	2022	Livestock handlers	Bov	AM	Ngov	[54]
	2022	Livestock handlers	Bov	AM	Ngov	[55]
United States	2012	n.a	Unspecified species	AM	Gov	[95]
	2020	Veterinarians	Unspecified species	AM	Gov	[105]

^a^OECD key partner and accession candidate

^b^OECD accession candidate

^c^OECD key partner

^d^This guideline is the same for Indonesia and Thailand, being accounted for only one; *Bov* bovine, *Cap* caprine, *Eq* equine, *Ov* ovine, *Sw* swine; Unspecified species: guidelines referred to ‘livestock’, ‘farm animals’, ‘production animals’, and/or ‘industrial animals’; Other species: other livestock animals (different than the main 5 addressed) and pets, including poultry, dogs, cats, fish, rabbits, chinchillas, hamster, deer, fur animals, bees, small rodents, New World camelids; *AM* antimicrobials, *AB* antibiotics; Livestock handlers: owners, farmers, producers, animal keepers, breeders, and other staff; Stakeholders: veterinarians, livestock handlers, pharmaceutical industries, health managers, regulatory authorities, laboratories, etc.; *Gov* governmental organization; *Ngov* non-governmental organization*; n.a.* not available

**Fig. 3:**
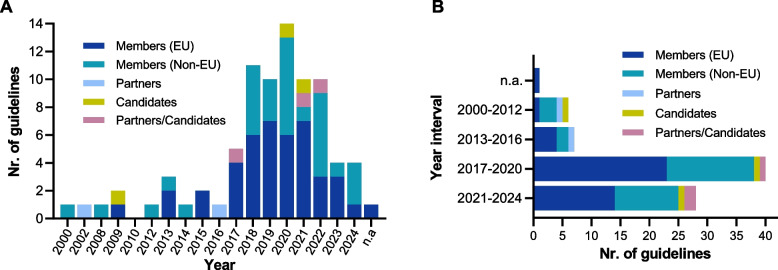
Number of guidelines published *per* year (**A**) and *per* year interval (**B**) based on OECD status. EU: European Union; n.a.: not available

As depicted in Fig. 3, most guidelines (*n* = 14, 17·07%) were published in 2020. From 2000 to 2016, 13 (15·85%) guidelines were found. Since 2017, the EU has published/updated 37 (45·12%), while other OECD countries have published/updated 31 (37·80%) guidelines. The publication year for the Slovenia guideline was not available (n.a.).

### Medically important antimicrobials

**Fig. 4:**
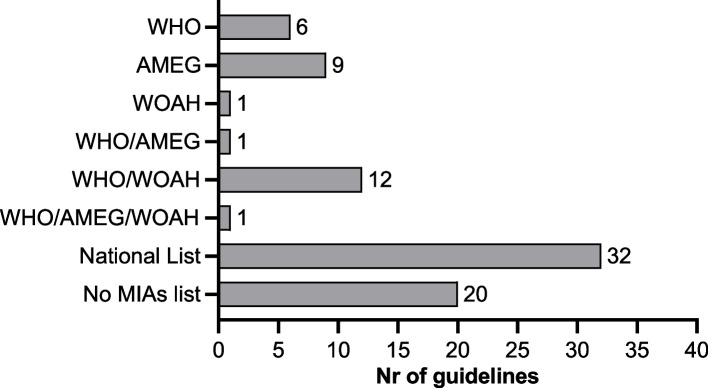
Number of guidelines that provide or indicate a list of medically important antimicrobials (MIAs). Within the national lists, 29 were based on the indicated international categorization and 3 did not specify any reference list. The specific organizations that established national lists for 22 guidelines are: Australian Strategic and Technical Advisory Group on Antimicrobial Resistance (ASTAG), Belgium’s Centre of Expertise on Antimicrobial Consumption and Resistance in Animals (AMCRA), Canada’s Veterinary Drug Directorate’s (VDD), the Danish Veterinary and Food Administration, Japan’s Ministry of Agriculture, Forestry and Fisheries (MAFF), Japan’s Food Safety Commission (FSC), the Netherlands Veterinary Antibiotic Policy Working Group (WVAB), the UK’s Pig Veterinary Society, and Veterinary Medicine Directorate (VMD); and the United States Food and Drug Administration (FDA); WHO: World Health Organization; AMEG: Antimicrobial Advice Ad Hoc Expert Group; WOAH: World Organisation for Animal Health; MIAs: medically important antimicrobials

Considering the importance of antimicrobials used in both human and veterinary medicine – MIAs – the number of guidelines that have or have directed the reader to a list of these critical antimicrobials was also reported (Fig. 4). Most of the guidelines either referred to the internationally established MIAs lists, WHO, AMEG (Antimicrobial Advice Ad Hoc Expert Group), and/or WOAH (*n* = 30) [26, 28, 33, 38, 45, 47, 49–55, 59, 70, 73, 78, 80, 81, 85–89, 92, 97, 98, 100], or presented national-specific lists based on those (*n* = 29). In the latter, 22 guidelines of 8 countries identified organizations that defined the antimicrobial classification [24, 25, 27, 29, 41, 42, 56, 58, 60, 64, 66, 68, 69, 71, 76, 80, 90, 91, 95, 103, 105, 119]. A total of 3 guidelines [46, 53, 75] also referenced MIAs, although not denoting any specific international classification. In turn, 20 guidelines did not mention MIAs [30–32, 34–37, 48, 62, 63, 65, 72, 78, 82–84, 94, 96, 99, 101].

Some differences were noted between country-specific and WHO’s lists, namely the classification as “low importance”, as opposed to the WHO’s CIA ranking . Also, some guidelines [95, 105] applied a higher importance to other antimicrobials, including the prohibition for use in food-producing animals or humans. Furthermore, additional antimicrobials beyond those provided by WHO have been considered as MIAs by several countries’ guidelines [39–42, 49, 56, 57, 59, 60, 66, 71, 74–76, 92, 93, 95, 100, 104, 105]. Detailed information regarding the active ingredients and the differences between these lists and WHO’s classification of MIAs can be found in the appendix (pp 12-16).

### Quality assessment of guidelines

**Fig. 5:**
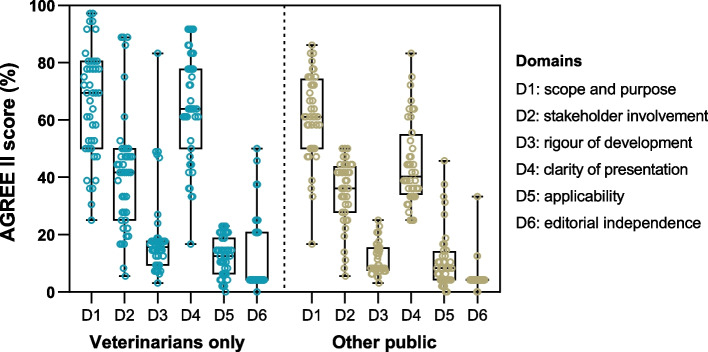
AGREE II domain scores, in percentage, for guidelines targeting either veterinarians or other public. Other public refers to guidelines that are non-clinic and addressed to veterinarians and/or other stakeholders

The quality appraisal using AGREE II revealed differences in some domain scores, depending on the nature of the guideline: clinical and non-clinical (Fig. 5). In both types of guidelines, the highest-scoring quality domains included scope and purpose, ranging from 25·00 to 97·22% and from 16·67 to 86·11%, for clinical and non-clinical guidelines, respectively. The clarity of presentation ranged from 16·67 to 91·67% and from 25·00 to 83·33%, for veterinarian-only and other public guidelines, respectively. Scores for stakeholder involvement ranged from 5·56 to 88·89% and from 5·56 to 50·00%, for clinical and non-clinical guidelines. Veterinarian-only guidelines showed a higher score for rigor of development (3·13 to 83·33%) compared to guidelines for other public (3·13 to 25·00%) (Fig. 5). The quality domain applicability ranged from 0·00 to 22·92% and 0·00 to 45·83% for veterinary-specific and other public guidelines, respectively. This suggests inadequate reporting on strategies to improve guideline uptake and on the implications and resources needed for implementation. Lastly, editorial independence ranged from 0·00 to 50·00% in veterinary-only guidelines and from 0·00 to 33·33% for documents directed towards other public. From the analyzed guidelines, only 2 (2·44%) disclosed that the formulation of recommendations was void of undue bias due to competing interests. More detailed information regarding AGREE II analysis is in the appendix (pp 17-21).

**Fig. 6:**
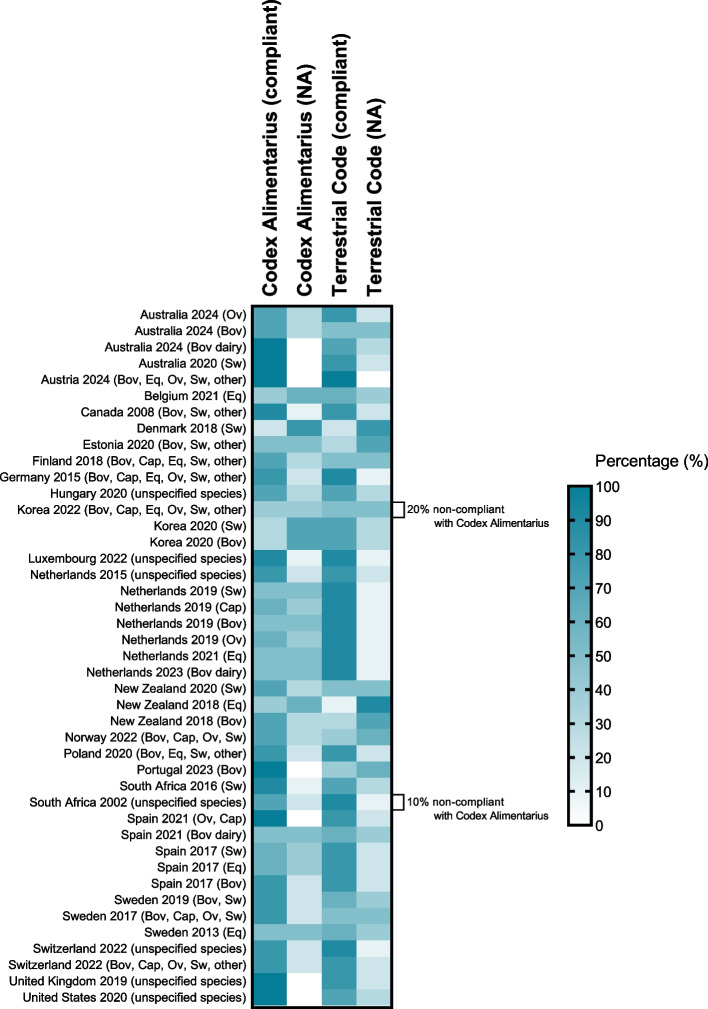
Compliance of recommendations with Codex Alimentarius (FAO/WHO) and Terrestrial Animal Health Code (WOAH). Guidelines were categorized as compliant when the recommendation was aligned with the document and as non-compliant when they were not. When the information was not found in the analyzed document, the recommendation was classified as Not Available (NA). The bar on the right depicts the color scale, ranging from 0 to 100%. The percentage of non-compliant recommendations is also indicated. For each international document, 10 recommendations were assessed. Details on the addressed topics are in appendix (pp 20-21)

Among the 43 clinical guidelines, a median of 70% (range of 20-100%) and 80% (range of 10-100%) compliance with the Codex Alimentarius and the Terrestrial Code, respectively, was observed. The median of non-available information regarding the listed recommendations was 30% (range of 0-80%) and 20% (range of 0-90%) for the Codex Alimentarius and the Terrestrial Code, respectively. Non-compliance with the Codex Alimentarius on the use of MIAs as growth promoters was only observed for Korea (20%) and South Africa (10%) guidelines (Fig. 6).

## Discussion

In this systematic review, we assessed the guidelines of 49 OECD-related countries on the prudent use of antimicrobials in food-producing animals. Our search retrieved 82 guidelines from official sources (and others identified by the government) from 37 countries, potentially hindering global efforts against AMR. Some countries only had guidelines directed towards producers and/or other stakeholders, despite veterinarians being primarily responsible for antimicrobial prescription and use, and in direct communication with farmers. Both clinical and non-clinical guidelines clearly defined the scope and purpose and displayed high clarity of presentation.

These guidance documents are part of the wider international antimicrobial stewardship framework, focusing on good husbandry practices and appropriate antimicrobial use as actions to reduce AMR emergence and spread. Results from antimicrobial stewardship initiatives are evident and measurable. For instance, antimicrobial sales for meat production halved in the last two decades across OECD countries [106] and, if the trend continues, antimicrobial use in food-producing animals could drop by an additional 10% by 2035 [106]. In Europe, the Nordic countries register some of the lowest use of antimicrobials in animals and AMR prevalence, outcomes of strict regulatory frameworks, guidelines’ application, surveillance programs, and adequate education and training [107, 108]. In Canada, MIAs quantity (mg/kg biomass) sold for use in animals decreased by 3% [109]. Antimicrobial stewardship relies on actively engagement of stakeholders, namely governmental agencies, provincial and territorial partners (Ministries of Agriculture and veterinary licensing bodies), veterinary medical associations, animal nutrition associations, drug manufacturers, as well as producer associations [110].

The One Health approach can only effectively mitigate AMR if strong collaborative efforts are in place. Therefore, each country should implement these guidelines according to their own regulatory framework, resources, and epidemiological status. Depending on these, some countries may require gradual adaptation and integration of guidelines. Nevertheless, countries have an increased understanding and awareness of AMR, showing commitment towards antimicrobial stewardship and helping each other. For instance, through the network “Nordic Vets Against AMR”, Finland, Norway, and Sweden aim to exchange knowledge and experiences to support other countries in effectively tackling AMR [108]. Noteworthy, contrary to the mandatory nature of antimicrobial use legislation, guidelines are voluntary to apply. Therefore, the ongoing improvement in food-producing practices and the responsible antimicrobial use demonstrates countries’ commitment to tackle AMR. Nations’ efforts are further proven by the increased publication of veterinary medicine guidelines after 2016 as part of NAPs. These strategic plans were promoted by WHO’s Global Action Plan on AMR, issued in 2015 [111]. The European Commission’s 2015 adoption of EU Guidelines on Prudent Use of Antimicrobials in Animal Health [112] may also explain this rise in guidelines publication. A peak in 2020 may be linked to Regulation (EU) 2019/6, effective in January 2019 [113], and the farm-to-fork strategy presented in May 2020 [114].

Moreover, commitment towards antimicrobial stewardship is demonstrated by guidelines that, besides addressing international recommendations, also included additional information and guidance, namely (i) euthanasia in specific cases (e.g.: uncertain prognosis, long-term disease, or if animal welfare cannot be ensured) [29, 32, 43–45, 50, 75, 103], (ii) decision-trees to instruct and guide on the choice of the most adequate antimicrobial [27, 62, 69, 75, 96], (iii) diagnostic protocols and a guide to interpret susceptibility test results [73], (iv) non-antibiotic alternatives for infection prevention in dry-period cows (e.g.: teat sealants) [54], (v) lists of disinfectants for cleaning animal housing and equipment, including their effectiveness and advantages/disadvantages [38, 55], (vi) the prescription is revoked and the treatment is terminated if the farmer does not address the deficiencies in husbandry conditions [50, 51], (vii) promote the implementation of new commercially available technologies for more cost-effective, quicker, and more accurate diagnosis (e.g.: of bovine respiratory disease) [25], and (viii) veterinarians are only allowed to prescribe and dispense under a valid veterinarian-client-patient-relationship (VCPR) [24, 25, 29, 64, 68, 78, 105]

In antimicrobial stewardship, the first and foremost step in the process of reducing antimicrobial use is the establishment of effective disease prevention measures, as highlighted in The One Health Joint Plan of Action (2022-2026) [115]. Among these are the adoption of appropriate biosecurity actions, vaccination plans, and management strategies, including pest control, housing, and nutrition [12, 106, 116]. Effective communication between the veterinarian and producer is key, as some producers may resist changes due to immediate financial burdens [117]. From 2013 to 2022, the global reduction in antimicrobial sales (20·9 to 12·7%) has been correlated with increased use of vaccines and parasiticides (56·7 to 62·8%) [118]. Most guidelines (*n* = 71) included biosecurity and preventive actions [24–32, 34–38, 41, 43–57, 59, 61–68, 70, 72, 73, 75–103, 105, 119].

The intensification of antimicrobial use in large-scale farming systems [120] is expected due to the increasing food needs of the growing global population [121, 122]. In intensive animal production systems, the occurrence and spread of infectious diseases is exacerbated when biosecurity is inadequate [123]. Therefore, antimicrobials are used routinely in these farming systems, sometimes at sub-therapeutic doses, not only to control sub-clinical infections and to maintain productivity, but also for prophylactic and metaphylactic purposes [120, 123]. In the EU, according to Regulation (EU) 2019/6, the use of all antimicrobials as productivity enhancers is prohibited and, in the case of metaphylaxis and prophylaxis, Article 107(3) states that antimicrobials are only allowed in specific conditions and when properly justified [113].

Since countries are becoming more interdependent and the OECD facilitates food trading, it is extremely important to outline and implement guidelines for the prudent use of antimicrobials in food-producing animals [124]. Recent trade agreements prioritize AMR, food sustainability, and animal welfare, namely EU-Mexico (2018) [125], UK-New Zealand (2022) [126], Australia-UK (2023) [127], EU-Chile (2023) [128], and EU-New Zealand (2024) [129]. A strategic association between Mercosur (Argentina, Brazil, Paraguay, Uruguay) and the EU, if ratified, could increase EU beef imports from Mercosur from 23 to 52% by 2030 [130] due to new tariff rate quotas [4, 130, 131]. Reserves are held regarding its approval, including increased agricultural, environmental, and health damage due to the misuse of pesticides and antibiotics prohibited in EU by Mercosur countries [130]. Despite Brazil and Argentina being key OECD partners or candidates, this negotiation highlights the difficulty in implementing standardized guidelines and the need for case-by-case analysis. Nevertheless, the EU and Mercosur have agreed to cooperate in ensuring animal welfare standards, food safety, and tackling AMR [132, 133].

Relating to MIAs, a considerable number of guidelines (24·39%) did not contain a MIAs list, and among those that had antimicrobial categorization, some differed from WHO’s list due to local conditions, low dependence on these drugs and/or the country-specific importance of some pathogens (e.g.: epidemiological status, legislation, and available resources) or required updated versions. Of note, since the first installment of MIAs categorization by WHO in 2005, the document has been updated seven times, with the most recent version being from 2024 [134]. Therefore, guidelines dating from previous years may have considered the classification that was in effect at the time. It is important to mention that WHO’s list of MIAs specifies that “the term ‘antimicrobial’ refers to antibacterials” and that “lists focused on other antimicrobials, such as antifungals, will be developed in the future to complement this list” [134]. Our results showed that more than half of the guidelines focused on antibacterial agents. However, caution is needed when interpreting this data. In some documents, the terms'antibacterial'and'antimicrobial'were used interchangeably without clear definition, suggesting confusion between the two. This raises concerns about the integrated approach to antimicrobial stewardship, particularly in recognizing the roles of all antimicrobial agents in the rise of AMR. It also emphasizes the need for clearer guidance and communication on AMR, which involves not only bacterial resistance, but also resistance in viruses, fungi, and protozoa.

Although not prohibited for use in animals, MIAs are strongly controlled in veterinary medicine in the EU under Regulation 2019/6 [113]. On 1 January 2006, the EU prohibited the use of all antibiotics for growth promotion [106] and, in February 2023, the EU adopted the Regulation (EU) 2023/905, banning imports of animal-derived products from third countries exposed to antimicrobials as growth promoters [135]. Some OECD countries still use non-MIAs for productivity purposes, namely Argentina, Australia, Brazil, India, Korea, South Africa, and the US [23, 136]. This occurs because farmers, in order to meet market demands and maintain their competitiveness, are required to increase production yields in a short period of time [137]. Also, regulatory framework regarding the use of antimicrobials for this purpose is often lacking, allowing their widespread availability without prescription [137, 138]. In South Africa, antimicrobials are available over-the-counter, and some MIAs are permitted as feed additives [139, 140]. Korea phased out antibiotics in compound feed in 2011 [141], but still allows farm-level mixing [141]. On 13 January 2020, Brazil banned growth promoters with tylosin, lincomycin, and tiamulin, but allows their production for export [142]. Nevertheless, OECD countries are progressively discouraging antimicrobials as yield enhancers. This is being accomplished by prohibiting antimicrobials as growth promoters [106, 143–147], by banning MIAs for productivity [136, 148, 149], or though the withdrawal of drug-containing feed additives [150].

For the successful implementation of recommendations, investment in information technologies and online platforms is important to increase the awareness of professionals and stakeholders, and to provide fast and easy access to guidelines. Countries can use digital technologies to facilitate consultation and application of recommendations. EPRUMA, a multi-stakeholder platform active since 2005, includes several European organizations and promotes good-practice frameworks for veterinary medicines [151]. In September 2023, the WOAH introduced ANIMUSE (ANImal antiMicrobial USE), a global interactive platform for reporting, accessing, and visualizing data on antimicrobial use in animals. This database presents global and regional data, helping countries assess the effectiveness of national measures and identify gaps [152]. In Belgium, in 2012, the AMCRA (AntiMicrobial Consumption and Resistance in Animals) created species-specific *vade-mecums*, i.e. pocketbooks or guides [104, 153], for the responsible antibacterial use, accessible via a website and a free app [104]. The UK’s Responsible Use of Medicines in Agriculture (RUMA) Alliance [154], the Netherlands Veterinary Medicines Institute (SDa) [155], and Spain’s Vet + i Foundation (Vetresponsable) [156] have websites dedicated to antimicrobials use in livestock for veterinarians and/or producers. Australia’s AMR Vet Collective provides guidance on antimicrobial prescription, communication with farmers, and offers online courses [157]. Canada’s Farmed Animal Antimicrobial Stewardship Initiative (FAAST) platform educates on antimicrobial use and collaborates with veterinarians, livestock owners, and stakeholders to prevent AMR dissemination [158].

However, not all online platforms ensure public accessibility. For example, Spain has an app for registered veterinarians, informing on recommended treatment by species and disease, antibiotic categorization, and the region’s epidemiological status [159]. Moreover, the Canadian Veterinary Medical Association (CVMA) launched the Firstline app (https://firstline.org/cvma/), a mobile repository of updated clinical guidance on antimicrobial use. Access to this resource is restricted to licensed Canadian veterinarians.

In human medicine, these online tools, namely mobile apps, have also demonstrated high applicability for clinical practice guidelines (CPGs) dissemination, easy access, and implementation by clinicians [160–164]. In addition, access to global CPGs is possible through the database Guidelines International Network [165], that encompasses a multitude of countries and languages, guidelines’ scope (disease, treatment/diagnosis, among others) and status (published, withdrawn, or planned), and endorsing organization [165].

In quality assessment using AGREE II, clinical and non-clinical guidelines scored high in domain 1 (aim, clinical question, and target population) and domain 4 (language, structure, and presentation), similar to other reports in veterinary [17] and human medicine [166, 167]. Nevertheless, both were lacking in rigor of development (domain 3), applicability (domain 5), and editorial independence (domain 6). The rigor of development, focusing on the process of identifying and summarizing evidence for guidelines’ development, scored poorly, indicating that either this information was omitted or the authors did not resort to established approaches to ensure methodological rigor [17, 166, 167]. Applicability also displayed a low score. This domain addresses aspects related to guidelines’ implementation and, if not adequately addressed, it may impair compliance [17, 166, 167]. A low applicability suggests that the guideline may not be as useful for clinicians as intended, requiring a design that considers not only scientific evidence, but also the dissemination and implementation for real-life use by clinicians [168]. The lowest scoring domain was editorial independence, which examines any conflicts of interest and the impact of funding entities. Appropriate declarations should be included in guidelines to ensure transparency. This domain’s scoring varies between animal and human health guidelines, being generally higher in the later [17, 166]. Stakeholder involvement had an intermediate score. This domain addresses if all the relevant individuals were included during the guidelines’ development and is highlighted as important in human [166, 169] and animal sectors [17]. Similar to the present study, recommendations in human medicine also vary widely across guidelines due to differences in target population and geographic location [170], leading to varied AGREE II scores [170, 171].

Overall, OECD and other countries worldwide are identifying gaps and working to promote the adequate use of antimicrobials in animals, either through regulatory frameworks, guiding documents, or both [106]. As aforementioned, disparities between nations/regions in terms of culture, internal policies, attitudes and perceptions about ABR, and financial resources must be accounted for when assessing antimicrobial stewardship initiatives. Importantly, the existence of national guidelines does not ensure their implementation or impact. Indeed, some countries in South America, Southeast Asia, and Africa, despite not yet fully integrating antimicrobial stewardship within their regulatory framework, are showing commitment to improving their practices and collaborate, at national and international levels, against AMR [172–174]. Nevertheless, these guidelines act as foundational pillars within a broader antimicrobial stewardship framework, providing countries with a structured reference to guide national efforts, align policies, and engage stakeholders.

## Strengths and limitations

Major strengths of this systematic review were the exhaustive search of antimicrobial guidelines on governmental and non-governmental entities. This allowed a broader understanding of current initiatives against AMR, emphasizing the need to improve coordination between countries and to encourage the design, dissemination, and implementation of appropriate and updated guidelines. Despite this, some limitations should be noted: (i) This study was focused on guidelines referenced on governmental websites or organizations designated by these entities and retrievable by the authors, i.e., without restricted access to members and/or veterinarians. Therefore, we recognize the limitations of the search strategy and the fact that some guidelines may not have been included. Nevertheless, guidelines officially endorsed or published by the government have been included and discussed. The non-inclusion of some documents may impact the comprehensiveness and accuracy of reported findings and limit their interpretation, with an underestimating of countries’ antimicrobial stewardship efforts and risking overly optimistic or pessimistic views of current national scenarios. However, this systematic review can serve as a source of in-depth knowledge and critical analysis of OECD countries’ guidelines for the prudent use of antimicrobials in food-producing animals; (ii) Throughout the guidelines’ assessment, the terms ‘antimicrobials’ and ‘antibiotics’ were sometimes used interchangeably, without explanation. This hindered the clear definition of what the focus of said guidelines was; (iii) In the absence of a tool to assess the quality of veterinary practice guidelines, the AGREE II instrument, recommended for human clinical practice guidelines, was used. Other authors have also employed this appraisal tool in the veterinary context, denoting its adaptability [17, 175]. Although this instrument is valuable for comparing guidelines, all domains have equal weight [22] and it has been considered subjective since there are no minimum domain scores set to define low- and high-quality [21]. Users may introduce different cut-offs, resulting in disparate ratings for the same guideline [176]. Moreover, there is no evidence correlating AGREE II domain scores with effective guideline implementation [22]. Another limitation of this tool is that the content of guidelines is not thoroughly accounted for, including the existence of online platforms/tools that provide guidance on antimicrobial use. This relevant item is not considered by this appraisal instrument; thus, several guidelines may be undervalued. In human clinical practice, it is recommended that two appraisers assess the guidelines [177]. In the present work, two appraisers, one a non-practicing veterinarian, performed this task. Nevertheless, a wider group of appraisers would be advantageous. Overall, the AGREE II scores showed the variability between guidelines, even within the same country, serving as a guiding foundation for which topics could benefit from improvement. It would be important to develop and validate a critical appraisal tool for veterinary medicine guidelines to ensure their quality, reliability, and applicability. Such tool could facilitate the integration of the latest research findings into veterinary practice and allow veterinarians to critically assess the validity and relevance of recommendations, leading to more informed clinical decisions.

## Conclusion

Global awareness about AMR is increasing, and consumer habits are shifting, favoring healthier food options and pressuring the agriculture sector to reduce drug use in livestock. This systematic review indicates that OECD-related countries are actively addressing AMR in food-producing animals or have proposed future strategies and interventions. For successfully designing and implementing guidelines, it is important to consider the heterogeneity in socio-economic and production systems across these countries. Since AMR prevalence varies by geography and antimicrobial class, appropriate surveillance is crucial for more targeted interventions. Investment in information technologies for guidelines’ dissemination should also be a priority. Overall, this systematic review can help policymakers identify potential gaps in current approaches, and design tailored and more effective stewardship programs from a One Health perspective, addressing drivers of AMR and mitigating this global health crisis.

## Supplementary Information

Additional file 1. Appendix: Additional material to: Evaluation of guidelines on antimicrobials use in food-producing animals: A systematic review; PDF format (.pdf); Includes the following information: Appendix 1: Searching terms strategy; Appendix 2: Template for guidelines’ data collection; Appendix Table S1: List of documents that did not meet the eligibility criteria and respective reasons for exclusion; Appendix Table S2: OECD countries and respective guidelines (last year of update and title) for the responsible and prudent use of antimicrobials in food-producing animals. When applicable, the translation of the title is within square brackets; Appendix Table S3: OECD countries without guidelines for the responsible use of antimicrobials in food-producing animals; Appendix Table S4: List of OECD countries and respective timeframe of established National Action Plans (NAPs) against AMR; Appendix Table S5: Lists of medically important antimicrobials (MIAs) in each guideline (*n* = 82); Appendix Table S6: AGREE II standardized scores by domain (D) for the 42 clinical guidelines considered in the quality appraisal; Appendix Table S7: Intraclass correlation coefficients, 95% confidence intervals, and interpretation for each AGREE II domain and item across the 42 appraised clinical guidelines; Appendix Figure S1: AGREE II standardized scores of all guidelines (clinical and non-clinical) by domain; Appendix Table S8: List of international recommendations from the Codex Alimentarius (*n* = 10) and from the Terrestrial Animal Health Code (*n* = 10) that were assessed for compliance in the retrieved guidelines; Appendix text S1: References.

## Data Availability

All data generated or analyzed during this study are included in this published article and its supplementary information file (Appendix).
