# Supplementary material for: Evaluation of guidelines on antimicrobials use in food-producing animals: a systematic review
Source: One Health Outlook. 2025 Aug 14;7:39. doi: 10.1186/s42522-025-00160-w (PMC12351981; doi:10.1186/s42522-025-00160-w)
Supplement: Supplementary file 1 — Additional file 1. Appendix: Additional material to: Evaluation of guidelines on antimicrobials use in food-producing animals: A systematic review; PDF format (.pdf); Includes the following information: Appendix 1: Searching terms strategy; Appendix 2: Template for guidelines’ data collection; Appendix Table S1: List of documents that did not meet the eligibility criteria and respective reasons for exclusion; Appendix Table S2: OECD countries and respective guidelines (last year of update and title) for the responsible and prudent use of antimicrobials in food-producing animals. When applicable, the translation of the title is within square brackets; Appendix Table S3: OECD countries without guidelines for the responsible use of antimicrobials in food-producing animals; Appendix Table S4: List of OECD countries and respective timeframe of established National Action Plans (NAPs) against AMR; Appendix Table S5: Lists of medically important antimicrobials (MIAs) in each guideline (n = 82); Appendix Table S6: AGREE II standardized scores by domain (D) for the 42 clinical guidelines considered in the quality appraisal; Appendix Table S7: Intraclass correlation coefficients, 95% confidence intervals, and interpretation for each AGREE II domain and item across the 42 appraised clinical guidelines; Appendix Figure S1: AGREE II standardized scores of all guidelines (clinical and non-clinical) by domain; Appendix Table S8: List of international recommendations from the Codex Alimentarius (n = 10) and from the Terrestrial Animal Health Code (n = 10) that were assessed for compliance in the retrieved guidelines; Appendix text S1: References. [file 42522_2025_160_MOESM1_ESM.docx]

**Appendix**

Additional material to: Evaluation of guidelines on antimicrobials use in food-producing animals: A systematic review.

Jacinta Oliveira Pinho^1,#^ PhD, Ana Isabel Plácido^2,#^ PhD, Alexandra Monteiro^2^ MSc, Rafaela Nogueira^3,4^ MSc, Paula Alexandra Oliveira^3,5^ PhD, Ana Claúdia Coelho^4,5,6^ PhD, Adolfo Figueiras^7,8^ PhD, Fátima Roque^2^ PhD, Maria Teresa Herdeiro^1^ PhD

^1^ Department of Medical Sciences, Institute of Biomedicine (iBiMED), University of Aveiro, Campus Universitário de Santiago, Agra do Crasto, 3810-193 Aveiro, Portugal

^2^ BRIDGES - Biotechnology Research, Innovation and Design for Health Products, Polytechnic University of Guarda, Avenida Dr. Francisco Sá Carneiro, n. 50, 6300-559 Guarda, Portugal

^3^ Centre for the Research and Technology of Agro-Environmental and Biological Sciences (CITAB), University of Trás-os-Montes and Alto Douro (UTAD), Vila Real, Portugal

^4^ Animal and Veterinary Research Centre (CECAV), Department of Veterinary Sciences, University of Trás-os-Montes and Alto Douro (UTAD), Vila Real, Portugal

^5^ Department of Veterinary Sciences, School of Agrarian and Veterinary Sciences, University of Trás-os-Montes and Alto Douro (UTAD), Vila Real, Portugal

^6^ Associate Laboratory for Animal and Veterinary Sciences (AL4AnimalS), Faculty of Veterinary Medicine, University of Lisboa, Lisboa, Portugal

^7^ Department of Preventive Medicine and Public Health, University of Santiago de Compostela; Health Research Institute of Santiago de Compostela (IDIS), Santiago de Compostela, Spain

^8^ Consortium for Biomedical Research in Epidemiology and Public Health (CIBER en Epidemiología y Salud Pública-CIBERESP), Carlos III Health Institute, Madrid, Spain

# Equally contributing authors

Correspondence to: Maria Teresa Herdeiro; [teresaherdeiro@ua.pt](mailto:teresaherdeiro@ua.pt)

**Table of Contents**

[Appendix 1: Searching terms strategy. 2](#_Toc196209394)

[Appendix 2: Template for guidelines’ data collection. 3](#_Toc196209395)

[Appendix Table S1: List of documents that did not meet the eligibility criteria and respective reasons for exclusion. 4](#_Toc196209396)

[Appendix Table S2: OECD countries and respective guidelines (last year of update and title) for the responsible and prudent use of antimicrobials in food-producing animals. When applicable, the translation of the title is within square brackets. 6](#_Toc196209397)

[Appendix Table S3: OECD countries without guidelines for the responsible use of antimicrobials in food-producing animals. 9](#_Toc196209398)

[Appendix Table S4: List of OECD countries and respective timeframe of established National Action Plans (NAPs) against AMR. 10](#_Toc196209399)

[Appendix Table S5: Lists of medically important antimicrobials (MIAs) in each guideline (n=82). 12](#_Toc196209400)

[Appendix Table S6: AGREE II standardized scores by domain (D) for the 42 clinical guidelines considered in the quality appraisal. 17](#_Toc196209401)

[Appendix Table S7: Intraclass correlation coefficients, 95% confidence intervals, and interpretation for each AGREE II domain and item across the 42 appraised clinical guidelines. 19](#_Toc196209402)

[Appendix Figure S1: AGREE II standardized scores of all guidelines (clinical and non-clinical) by domain. 21](#_Toc196209403)

[Appendix Table S8: List of international recommendations from the Codex Alimentarius (n=10) and from the Terrestrial Animal Health Code (n=10) that were assessed for compliance in the retrieved guidelines. 22](#_Toc196209404)

[Appendix text S1: References. 24](#_Toc196209405)

# **Appendix 1: Searching terms strategy.**

These terms were translated to each OECD country’s original language and searched using the search engines provided in the official websites of the governmental bodies of the 49 countries investigated in our study.

“antimicrobial”

“antibiotics”

“AMR”

“bacteria”

“antibacterial resistance”

“antimicrobial resistance”

“veterinary guidance”

“veterinary guidelines”

“veterinary recommendations”

**Population:** All stakeholders involved in developing, revising, and implementing guidelines for the responsible and prudent use of antimicrobials in food-producing animals.

**Intervention:** Prescription of antimicrobials in veterinary, specifically in food-producing animals.

**Comparator:** International recommendations on antimicrobial use, as established by WHO, WOAH, and FAO: Codex Alimentarius: Code of Practice to Minimize and Contain Foodborne Antimicrobial Resistance, the Terrestrial Animal Health Code, and WHO Guidelines on Use of Medically Important Antimicrobials In Food-Producing Animals

**Outcome:** Existence/non-existence of guidelines, their specificities, and agreement/disagreement with international recommendations.

# **Appendix 2: Template for guidelines’ data collection.**

| **General characteristics of guidelines** | | | | | | | | |
| --- | --- | --- | --- | --- | --- | --- | --- | --- |
| Country | EU member (Yes/No) | Guideline (title) | Last update (year) | Target audience | Target species | Antimicrobial (AM)/ Antibiotic (AB) | Governmental organization (Gov)/ non-governmental organization (ngov) | Medically Important Antimicrobials (MIAs; existence/non-existence of lists; drugs’ name and classification) |
|  |  |  |  |  |  |  |  |  |

| **Agreement/disagreement with international guidelines** | |
| --- | --- |
| **INTERNATIONAL GUIDELINES** | [Country's Guideline] |
| **Codex Alimentarius: CODE OF PRACTICE TO MINIMIZE AND CONTAIN FOODBORNE ANTIMICROBIAL RESISTANCE CXC 61-2005 FAO, 2021** | *Does the guideline address these topics? [Yes/No]* |
| Biosecurity, appropriate nutrition, vaccination, animal and plant/ crop best management practices, and other alternative tools where appropriate, should be considered to reduce the need for use of antimicrobial agents. |  |
| The decision to use antimicrobial agents should be based on sound clinical judgement, experience, and treatment efficacy. |  |
| Medically important antimicrobials should be prescribed, administered, or applied only by, or under the direction of, veterinarians. |  |
| Antimicrobial agents should be used as legally authorized and following all applicable label directions; except where specific legal exemptions apply. |  |
| The choice of which antimicrobial agent to use should take into consideration relevant professional guidelines, where available, results of antimicrobial susceptibility testing of isolates from the production setting, where appropriate, and make adjustments to the antimicrobial agent selection based on clinical outcomes or when foodborne AMR risks become evident. |  |
| Responsible and prudent use of antimicrobial agents does not include the use for growth promotion of antimicrobial agents that are considered medically important. |  |
| Medically important antimicrobials should only be administered or applied for prevention/prophylaxis where professional oversight has identified well-defined and exceptional circumstances, appropriate dose and duration, based on clinical and epidemiological knowledge, consistent with the label, and in line with national legislation. |  |
| When used for the control of disease/metaphylaxis, medically important antimicrobial agents should only be used on the basis of epidemiological and clinical knowledge and a diagnosis of a specific disease and follow appropriate professional oversight, dose, and duration. |  |
| Countries could use additional risk management measures for medically important antimicrobials considered highest priority critically important as described in the WHO List of Critically Important Antimicrobials for Human Medicine, the OIE List of Antimicrobial Agents of Veterinary Importance, or national lists, where available, including restrictions proportionate to risk and supported by scientific evidence. |  |
| Monitoring and surveillance of the use of antimicrobial agents and the incidence or prevalence, and in particular trends, of foodborne AMR microorganisms and resistance determinants are among the critical factors to consider when developing risk management measures and evaluating the effectiveness of implemented risk management measure |  |
| **Terrestrial Animal Health Code (2023) Volume 1 CHAPTER 6.10. Responsible and prudent use of antimicrobial agents in veterinary medicine** |  |
| Administer or prescribe antimicrobial agents only when necessary and taking into consideration the WOAH list of antimicrobial agents of veterinary importance; |  |
| Make an appropriate choice of antimicrobial agents based on clinical experience and diagnostic laboratory information (pathogenic agent isolation, identification and antibiogram) where possible; |  |
| Provide a detailed treatment protocol, including precautions and withdrawal times, especially when prescribing extra-label or off-label use. |  |
| 2. Choosing antimicrobial agents |  |
| a) The expected efficacy of the treatment is based on: |  |
| i) the clinical experience of the veterinarians, their diagnostic insight and therapeutic judgement; |  |
| ii) diagnostic laboratory information (pathogenic agent isolation, identification and antibiogram); |  |
| iii) pharmacodynamics including the activity towards the pathogenic agents involved; |  |
| iv) the appropriate dosage regimen and route of administration; |  |
| v) pharmacokinetics and tissue distribution to ensure that the selected therapeutic agent is effective at the site of infection; |  |
| vi) the epidemiological history of the rearing unit, particularly in relation to the antimicrobial resistance profiles of the pathogenic agents involved. |  |
| Should a first-line antimicrobial treatment fail or should the disease recur, a second line treatment should be based on the results of diagnostic tests. In the absence of such results, an appropriate antimicrobial agent belonging to a different class or sub-class should be used. |  |
| In emergencies, a veterinarian may treat animals without recourse to an accurate diagnosis and antimicrobial susceptibility testing, to prevent the development of clinical disease and for reasons of animal welfare. |  |
| b) Use of combinations of antimicrobial agents should be scientifically supported. Combinations of antimicrobial agents may be used for their synergistic effect to increase therapeutic efficacy or to broaden the spectrum of activity. |  |
| A prescription for VMP containing antimicrobial agents should indicate precisely the dosage regimen, the withdrawal period (where applicable) and the amount of VMP containing antimicrobial agents to be provided, depending on the dosage and the number of animals to be treated.) |  |
| The extra-label or off-label use of VMP containing antimicrobial agents may be permitted in appropriate circumstances and should be in agreement with the national legislation in force including the withdrawal periods to be used, as applicable. It is the veterinarian's responsibility to define the conditions of responsible use in such a case including the dosage regimen, the route of administration and the withdrawal period. |  |

# **Appendix Table S1: List of documents that did not meet the eligibility criteria and respective reasons for exclusion.**

| **Country** | **Year** | **Name** | **Reason** |
| --- | --- | --- | --- |
| Argentina | 2017 | Pautas para el uso prudente de antimicrobianos en bovinos [Guidelines for the prudent use of antimicrobials in cattle] | Not an antimicrobial stewardship guideline* |
| Belgium | 2021 | Utilisation Raisonnée des Antibiotiques au Tarissement chez la Vache Laitière en Belgique [Reasonable use of Antibiotics for Drying Up at the Dairy Cow in Belgium] | Document specific to a procedure/treatment/active substance/disease. |
|  | 2024 | Lignes Directrices pour un Usage Prudent du Florfénicol chez les Animaux [Guidelines for Careful use of Florfenicol in Animals] | Document specific to a procedure/treatment/active substance/disease. |
| China | 2021 | 农业农村部关于印发《全国兽用抗菌药使用减量化行动方案（2021–2025年）》的通知 [Notice from the Ministry of Agriculture and Rural Affairs on the issuance of the "National Action Plan for Reducing the Use of Veterinary Antimicrobial Drugs (2021-2025)"] | Not an antimicrobial stewardship guideline* |
| Colombia | 2020 | Manual de Condiciones de Bienestar Animal propias de cada una de las especies de producción del sector agropecuario; bovina, bufalina, aves de corral y animales acuáticos [Manual of Animal Welfare Conditions specific to each of the production species in the agricultural sector; bovine, buffalo, poultry and aquatic animals] | Lack of information pertinent to the study † |
|  | 2020 | Manual de Condiciones de Bienestar Animal propias de cada una de las especies de producción en el Sector Agropecuario para las especies Équidas, Porcinas, Ovinas y Caprinas [Manual of Animal Welfare Conditions specific to each one of the production species in the Agricultural Sector for Equidae, Swine, Sheep and Goat species] | Lack of information pertinent to the study † |
| Costa Rica | 2018 | Manual de buenas prácticas en la producción de cerdo [Manual of good practices in pork production] | Lack of information pertinent to the study † |
| Czechia | 2014 | Veterinary medicinal product under prudent use | Lack of information pertinent to the study † |
|  | 2017 | Příručka pro faremní a řemeslné zpracování mléka [Handbook for farm and craft milk processing] | Not an antimicrobial stewardship guideline* |
|  | 2022 | Volby Antimikrobiálních Látek Pro Racionální Léčbu Infekčních Onemocnění Hospodářských Zvířat (Skotu, Prasat A Drůbeže) [Choices of Antimicrobial Agents for Rational Treatment of Infectious Diseases of Farm Animals (cattle, pigs and poultry)] | Document specific to a procedure/treatment/active substance/disease. |
| Denmark | n.a. | Kriterier for valg af antibiotika til kvæg [Criteria for choosing antibiotics for cattle] | Lack of information pertinent to the study † |
|  | 2020 | “God Klinisk Praksis” for praktiserende dyrlægers virke i svinebesætninger specielt med henblik på flokmedicinering [“Good Clinical Practice” for the work of practicing veterinarians in pig herds, especially with a view to herd medication] | Lack of information pertinent to the study † |
|  | 2022 | Anbefaling om metafylaktisk behandling af produktionsdyr [Recommendation on metaphylactic treatment of production animals] | Document specific to a procedure/treatment/active substance/disease. |
| Germany | 2010 | Guidelines for the prudent use of veterinary antimicrobial drugs – with notes for guidance | Non-updated version |
| Hungary | 2019 | Az antibiotikumok engedélyezésének és felhasználásának jogszabályi háttere [Legal background for the authorization and use of antibiotics] | Not an antimicrobial stewardship guideline* |
| Iceland | n.a. | Leiðbeiningar Ábyrg notkun sýklalyfja við slefsýki í lömbum [Guidelines for the responsible use of antibiotics for drooling in lambs] | Document specific to a procedure/treatment/active substance/disease. |
| India | 2014 | Use of antibiotics in food-producing animals No. 102-74/2014 | Not an antimicrobial stewardship guideline* |
|  | 2019 | Advisory – Rational use of antibiotics for limiting antimicrobial resistance (F. No. AMR/Misc/02/NCDC-NAP-AMR/18) | Not an antimicrobial stewardship guideline* |
| Ireland | 2022 | Prudent Prescribing of Dry-Cow and In-Lactation Antibiotics - Guidelines for PVPs | Document specific to a procedure/treatment/active substance/disease. |
| New Zealand | 2017 | Prudent Use of Antimicrobials on Animals and Plants | Not an antimicrobial stewardship guideline* |
| Norway | 2016 | Antibiotika - råd og retningslinjer for bruk hos dyr [Antibiotics - advice and guidelines for use in animals] | Lack of information pertinent to the study † |
| Poland | 2017 | Materiały informacyjne dla lekarzy weterynarii wolnej praktyki dotyczące zasad prowadzenia dokumentacji lekarsko-weterynaryjnej oraz dokumentacji obrotu detalicznego produktami leczniczymi weterynaryjnymi [Informational materials for free practice veterinarians on the principles of veterinary medical record keeping and retail product trading records veterinary medicines] | Not an antimicrobial stewardship guideline* |
| Slovenia | n.a. | Priporočila za uporabo antibiotikov [Recommendations for the use of antibiotics] | Document specific to a procedure/treatment/active substance/disease |
| South Africa | 2016 | Colistin use by veterinarians | Document specific to a procedure/treatment/active substance/disease. |
| Sweden | 2020 | “The Swedish experience” – a summary on the Swedish efforts towards a low and prudent use of antibiotics in animal production | Not an antimicrobial stewardship guideline* |
| Thailand | 2020 | กรมทรัพยากรธรรมชาติและสิ่งแวดล้อม บริหารจัดการงานตามแผนบริหารจัดการปราบปรามยาต้านเชื้อแบคทีเรียของประเทศไทย พ.ศ. 2560-2564 [The Department of Natural Resources and Environment manages work under the management plan for antibacterial drug suppression in Thailand 2017-2021] | Not an antimicrobial stewardship guideline* |
| Türkiye | 2017 | Tüba-insan ve hayvan sağlığında akılcı antibiyotik kullanımı ve antibiyotik dirençlilik raporu [tüba-rational antibiotic in human and animal health use and antibiotic resistance report] | Not an antimicrobial stewardship guideline* |
| * This includes regulatory documents, reports, national action plans (NAPs), scientific oral communication, notification/notice/advisory document, guidance on food processing practices for farmers, articles; n.a.: not available; † Does not address the prudent use of antimicrobials, refers to veterinary medicines in general, and/or only provides general information. | | | |

# **Appendix Table S2: OECD countries and respective guidelines (last year of update and title) for the responsible and prudent use of antimicrobials in food-producing animals. When applicable, the translation of the title is within square brackets.**

| Country | Last Update | Antimicrobial Guideline | References |
| --- | --- | --- | --- |
| Australia | 2020 | Antimicrobial prescribing guidelines for pigs | [1] |
|  | 2024 | Antimicrobial prescribing guidelines for sheep | [2] |
|  | 2024 | Antimicrobial prescribing guidelines for dairy cattle | [3] |
|  | 2024 | Antimicrobial prescribing guidelines for feedlot cattle | [4] |
| Austria | 2024 | Leitlinien für den sorgfältigen Umgang mit antibakteriell wirksamen Tierarzneimitteln [Guidelines for the careful handling of antibacterial veterinary medicinal products] | [5] |
| Belgium | 2021 | Mesures pour un bon usage des antibiotiques lors d’un traitement de groupe chez les bovins [Measures for the correct use of antibiotics during group treatment of bovines] | [6] |
|  | 2021 | Mesures pour un bon usage des antibiotiques lors d’un traitement de groupe des porcs [Measures for the correct use of antibiotics during group treatment of pigs] | [7] |
|  | 2021 | Surveillance et utilisation des antibiotiques, y compris de ceux d’importance critique, chez les chevaux [Monitoring and use of antibiotics, including those of critical importance, in horses. Current situation and recommendations for the future.] | [8] |
| Brazil‡ | 2022 | Atualização sobre Uso Racional de Antimicrobianos e Boas Práticas de Produção [Update on the Rational Use of Antimicrobials and Good Production Practices] | [9] |
| Bulgaria* | 2021 | Ръководство за разумна и отговорна употреба на антимикробни средства във ветеринарната медицина и практика [Guidelines for the reasonable and responsible use of antimicrobial agents in veterinary medicine and practice] | [10] |
| Canada | 2008 | Antimicrobial Prudent Use Guidelines for beef cattle, dairy cattle, poultry, and swine | [11] |
| Colombia | 2019 | Manual de Buenas Prácticas en el uso de Medicamentos Veterinarios en la Porcicultura [Manual for Good Practices for the use of Veterinary Medicines in Pig Farming] | [12] |
|  | 2020 | Guía de uso prudente de antibióticos en la producción de leche a partir del modelo de salud de hato [Guide to the prudent use of antibiotics in milk production from the herd health model] | [13] |
| Costa Rica | 2018 | Guía de uso responsable de medicamentos veterinarios en bovinos [Guide for the responsible use of veterinary medicines in bovines] | [14] |
| Denmark | 2013 | Retningslinjer for brug af antibiotika til kvæg i Danmark [Guidelines for the use of antibiotics for cattle in Denmark] | [14] |
|  | 2018 | Guideline for Prescribing Antimicrobial for Pigs | [15] |
| Estonia | 2020 | Juhend Antibiootikumide Kasutamiseks Põllumajandusloomadel (Clinical guidelines, clinical practice guidelines) [Guidelines for the use of Antibiotics in Farm Animals (Clinical guidelines, clinical practice guidelines)] | [16] |
| Finland | 2018 | Recommendations for the use of antimicrobials in the treatment of the most significant infectious and contagious diseases in animals | [17] |
| France | 2009 | Guide de bonnes pratiques de l’antibiothérapie vétérinaire à l’usage des vétérinaires [Guide to good practice in veterinary antibiotic therapy for veterinarians] | [18] |
| Germany | 2015 | Guidelines for the prudent use of veterinary antimicrobial drugs -with notes for guidance- | [19] |
| Greece | 2020 | Εγχειριδιο εμποριασ, προμηθειασ και χρησησ κτηνιατρικων φαρμακευτικων προϊοντων [Handbook on marketing, supply and use of veterinary medicinal products] | [20] |
| Hungary | 2020 | Az antibiotikum- kezelés minimumkövetelményeire vonatkozó útmutató [Guidelines for minimum requirements for antibiotic treatment] | [21] |
| Indonesia‡ | 2017 | ASEAN Guidelines for the Prudent Use of Antimicrobial In Livestock | [22] |
|  | 2021 | Pedoman Umum - Penggunaan Antibiotik di Bidang Peternakan dan Kesehatan Hewan [General Guidelines - Use of Antibiotics in the Field of Animal Husbandry and Animal Health] | [23] |
| Ireland | 2018 | Code of Good Practice Regarding the Responsible Prescribing and Use of Antibiotics in Farm Animal | [24] |
|  | 2019 | Code of Good Practice Regarding the Responsible Use of Antimicrobials on Pig Farms | [25] |
|  | 2019 | Code of Good Practice Regarding the Responsible Use of Antimicrobials on Dairy Farms | [26] |
|  | 2020 | Code of Good Practice regarding responsible use of Antimicrobials on Suckler and Beef Farms | [27] |
|  | 2020 | Code of Good Practice Regarding the Responsible Use of Antimicrobials on Sheep Farms | [28] |
|  | 2022 | Equine Antimicrobial Use Guidelines | [29] |
| Italy | 2018 | Vademecum per un uso prudente e razionale degli antibiotici nelle produzioni zootecniche [Vademecum for the prudent and rational use of antibiotics in livestock production] | [30] |
|  | 2018 | Linee guida per l'uso prudente degli antimicrobici negli allevamenti zootecnici per la prevenzione dell’antimicrobico-resistenza e proposte alternative [Guidelines for the prudent use of antimicrobials in zootechnical farming for the prevention of antimicrobial resistance and alternative proposals] | [31] |
|  | 2022 | Linee guida - Uso prudente degli antibiotici nell’allevamento suino [Guidelines - Prudent use of antibiotics in pig farming] | [32] |
|  | 2023 | Linee guida - Uso prudente dell'antibiotico nell'allevamento bovino da latte [Guidelines Prudent use of antibiotics in dairy cattle breeding] | [33] |
| Japan | 2000 | 畜産物生産における動物用抗菌性物質製剤の 慎重使用に関する基本的な考え方 [Basic concept regarding the prudent use of animal antibacterial preparations in livestock product production] | [34] |
|  | 2013 | 動物用抗菌剤の 『責任ある慎重使用』を 進めるために [To promote the responsible and prudent use of veterinary antimicrobial agents] | [35] |
| Korea | 2020 | 소 항생제 처방 가이드라인 [Bovine antibiotic prescribing guidelines] | [36] |
|  | 2020 | 돼지 항생제 처방 가이드라인 [Swine antibiotic prescribing guidelines] | [37] |
|  | 2022 | 산업동물 수의사를 위한 항생제 길잡이 [Antibiotic Guide for Veterinarians of Industrial Animals] | [38] |
| Latvia | 2018 | Vadlīnijas antimikrobiālās rezistences attīstības ierobežošanai lauksaimnieciskās ražošanas posmā un veterinārmedicīnas praksē Latvijā [Guidelines for limiting the development of antimicrobial resistance during agricultural production in veterinary practice in Latvia] | [39] |
| Luxemburg | 2022 | Empfehlungen für den sachgemäßen Einsatz von Antibiotika in der Tiergesundheit [Recommendations for the appropriate use of antibiotics in animal health] | [40] |
| Mexico | 2021 | Guía para el Buen Uso de Productos Farmacéuticos Veterinarios [Guide for the Good Use of Veterinary Pharmaceutical Products] | [41] |
|  | 2023 | Guía Del Buen Uso De Antimicrobianos En Cerdos [Guide to the Good Use of Antimicrobials in Pigs] | [42] |
| Netherlands | 2015 | Richtlijn Toepassen van antimicrobiële middelen [Guideline for the use of antimicrobials] | [43] |
|  | 2019 | Werkgroep Vterinair Antibioticumbeleid - Formularium Varken [Working Group on Veterinary Antibiotic Policy - Pig Formulary] | [44] |
|  | 2019 | Werkgroep Vterinair Antibioticumbeleid - Formularium Geit [Veterinary Antibiotic Policy Working Group - Goat Formulary] | [45] |
|  | 2019 | Formularium Vleeskalveren en Vleesvee [Formulary for veal calves and beef cattle] | [46] |
|  | 2019 | Formularium Kleine Herkauwers - Schaap [Formulary of small ruminants - sheep] | [47] |
|  | 2021 | Werkgroep Vterinair Antibioticumbeleid - Formularium Paard [Working Group on Veterinary Antibiotic Policy - Horse Formulary] | [48] |
|  | 2023 | Werkgroep Vterinair Antibioticumbeleid - Formularium Melkvee [Working Group on Veterinary Antibiotic Policy - Dairy Cattle Formulary] | [49] |
| New Zealand | 2018 | Antibiotic judicious use guidelines for the New Zealand veterinary profession in Dairy | [50] |
|  | 2018 | Guide to prudent use of antimicrobial agents in red meat production | [51] |
|  | 2018 | Antibiotic judicious use guidelines for the New Zealand veterinary profession in Equine | [52] |
|  | 2020 | Guide to prudent use of antimicrobial agents in Pigs | [53] |
| Norway | 2022 | Terapianbefaling: Bruk av antibakterielle midler til produksjonsdyr [Therapeutic recommendation: Use of antibacterial agents for production animals] | [54] |
| Poland | 2020 | Kodeks Rozważnego Stosowania Produktów Leczniczych Przeciwdrobnoustrojowych Przez Lekarzy Weterynarii [Code of the prudent use of antimicrobial medicinal products by veterinarians] | [55] |
| Portugal | 2021 | Manual de Boas Práticas Utilização de Antimicrobianos em Animais Produtores de Géneros Alimentícios [Manual of Good Practices for the Use of Antimicrobials in Food-Producing Animals] | [56] |
|  | 2023 | Utilização prudente de antimicrobianos em animais - Vacas leiteiras [Prudent use of antimicrobials in animals dairy cows] | [57] |
| Romania* | 2020 | Ghidul naţional privind utilizarea prudentă a antimicrobienelor în medicina veterinară [National guidelines on the prudent use of antimicrobial in veterinary medicine] | [58] |
| Slovenia | n.a. | Splošna priporočila za uporabo antibiotikov v veterinarske namene [General recommendations for the use of antibiotics for veterinary purposes] | [59] |
| South Africa† | 2002 | Technical Guidelines for Responsible and Prudent Use of Antimicrobials in Veterinary Medicine in South Africa | [60] |
|  | 2016 | Guidelines for the use of antimicrobials in the South African pig industry | [61] |
| Spain | 2017 | Guía de Uso Responsable de Medicamentos Veterinarios: Bovino [Guide for the Responsible Use of Veterinary Medicines: Bovine] | [62] |
|  | 2017 | Guía de Uso Responsable de Medicamentos Veterinarios: Equinos [Guide for the Responsible Use of Veterinary Medicines: Equines] | [63] |
|  | 2017 | Guía de Uso Responsable de Medicamentos Veterinarios: Porcino [Guide for the Responsible Use of Veterinary Medicines: Porcine] | [64] |
|  | 2021 | Recomendaciones para un uso prudente de los antibióticos en ganado bovino lechero [Recommendations for prudent use of antibiotics in dairy cattle] | [65] |
|  | 2021 | Guía de Uso Responsable de Medicamentos Veterinarios: Ovino y caprino [Guide for the Responsible Use of Veterinary Medicines: Sheep and goats] | [66] |
| Sweden | 2013 | The Swedish Veterinary Association’s Guidelines for the clinical use of antibiotics in the treatment of horses | [67] |
|  | 2017 | Guidelines for the use of antibiotics in production animals - Cattle, pigs, sheep and goats | [68] |
|  | 2019 | Sveriges Veterinärförbunds Riktlinjer För Antibiotikaanvändning Till Nötkreatur & Gris [Swedish Veterinary Association Guidelines for Antibiotic Use in Cattle and Pigs] | [69] |
| Switzerland | 2022 | Guide thérapeutique pour les vétérinaires - Utilisation prudente des antibiotiques: Bovins, Porcs, Petits Ruminants et Camélidés du Nouveau Monde [Therapeutic guide for veterinarians - Prudent use of antibiotics: cattle, pigs, small ruminants and New World camelids] | [70] |
|  | 2022 | Directives concernant l'emploi judicieux des médicaments vétérinaires [Guidelines for the judicious use of veterinary drugs] | [71] |
| Thailand* | 2009 | Code Of Practice for Control of the Use of Veterinary Drugs | [72] |
|  | 2017 | ASEAN Guidelines for the Prudent Use of Antimicrobial in Livestock | [22] |
| United Kingdom | 2014 | Code of Practice on the responsible use of animal medicines on the farm | [73] |
|  | 2018 | Practical Guide to Responsible Use of Antibiotics on Pig Farms | [74] |
|  | 2019 | BVA policy position on the responsible use of antimicrobials in food producing animals | [75] |
|  | 2019 | Industry guidance document for veterinary surgeons and farmers on responsible use of antibiotics in sheep | [76] |
|  | 2020 | Using medicines responsibly: As little as possible, but as much as necessary | [77] |
|  | 2022 | Guidelines on Responsible Use of Antimicrobials in Dry Cow Strategies | [78] |
|  | 2022 | Guidelines for Responsible use of antimicrobials in cattle production | [79] |
| United States | 2012 | The Judicious Use of Medically Important Antimicrobial Drugs in Food-Producing Animals | [80] |
|  | 2020 | Guidelines for Veterinarians: Judicious Use of Antimicrobials in Livestock; Guidance for Industry | [81] |
| Notes: * Candidates to the OECD; † Key partners of the OECD; ‡ Key partners and accession candidates of the OECD; n.a.: not available. | | | |

# **Appendix Table S3: OECD countries without guidelines for the responsible use of antimicrobials in food-producing animals.**

| **Country** | **OECD status** | **Geographical region** | **Income ^(a)^** | **Developed/Developing Country ^(b)^** |
| --- | --- | --- | --- | --- |
| Argentina | Candidate | Latin America | UMI | Developing |
| Chile | Member | Latin America | HIC | Developing |
| China | Key partner | Asia | UMI | Developing |
| Croatia | Candidate | Europe | HIC | Developed |
| Czechia | Member | Europe | HIC | Developed |
| Iceland | Member | Europe | HIC | Developed |
| India | Key partner | Asia | LMI | Developing |
| Israel | Member | Asia | HIC | Developed |
| Lithuania | Member | Europe | HIC | Developed |
| Peru | Candidate | Latin America | UMI | Developing |
| Slovakia | Member | Europe | HIC | Developed |
| Türkiye | Member | Asia | UMI | Developing |
| (a) The World Bank. The World by Income and Region. 2023. <https://datatopics.worldbank.org/world-development-indicators/the-world-by-income-and-region.html> (accessed July 11 2024).  (b) OECD/FAO. Agricultural Outlook 2024-2033. OECD Publishing, Paris: OECD; 2024. | | | | |

# **Appendix Table S4: List of OECD countries and respective timeframe of established National Action Plans (NAPs) against AMR.**

| **Country** | **OECD status** | **NAP timeframe** | **Guidelines for antimicrobial use in food-producing animals** | **Link to Source** |
| --- | --- | --- | --- | --- |
| Argentina | Candidate | 2022-2025 | No | [Argentina NAP](https://www.argentina.gob.ar/normativa/nacional/resoluci%C3%B3n-2291-2023-390855/texto) |
| Australia | Member | 2023-2028 | Yes | [Australia NAP](https://www.agriculture.gov.au/about/news/australias-anti-microbial-action-plan) |
| Austria | Member | 2023 (published) | Yes | [Austria NAP](https://www.sozialministerium.at/dam/jcr:685ce623-4e33-4f34-a77f-1b9786381a73/NAP-AMR_Kurzfassung.pdf) |
| Belgium | Member | 2020-2024 | Yes | [Belgium NAP](https://www.health.belgium.be/sites/default/files/uploads/fields/fpshealth_theme_file/fr-amr_one_health_national_plan_final.pdf) |
| Brazil | Key partner/Candidate | 2023-2027 | Yes | [Brazil NAP](https://www.gov.br/agricultura/pt-br/assuntos/insumos-agropecuarios/insumos-pecuarios/resistencia-aos-antimicrobianos/pan-br-agro/PlanodeAoda2EtapadoPANBRAGROjun.23.pdf) |
| Bulgaria | Candidate | 2023-2027 | Yes | [Bulgaria NAP](https://www.sp2023.bg/index.php/bg/component/dpattachments/?view=attachment&tmpl=component&id=75) |
| Canada | Member | 2023-2027 | Yes | [Canada NAP](https://www.canada.ca/content/dam/phac-aspc/documents/services/publications/drugs-health-products/pan-canadian-action-plan-antimicrobial-resistance/building-momentum-activities-underway-address-antimicrobial-resistance-canada.pdf) |
| Chile | Member | 2021-2025 | No | [Chile NAP](https://diprece.minsal.cl/wp-content/uploads/2023/10/2023.08.29_PLAN-OPERATIVO-RESISTENCIA-ANTIMICROBIANOS.pdf) |
| China | Key partner | 2022-2025 | No | [China NAP](http://www.nhc.gov.cn/yzygj/s7659/202210/2875ad7e2b2e46a2a672240ed9ee750f.shtml) |
| Colombia | Member | 2018 (published) | Yes | [Colombia NAP](https://www.minsalud.gov.co/sites/rid/Lists/BibliotecaDigital/RIDE/VS/MET/plan-respuesta-resistencia-antimicrobianos.pdf) |
| Costa Rica | Member | 2018-2025 | Yes | [Costa Rica NAP](https://faolex.fao.org/docs/pdf/cos181660.pdf) |
| Croatia | Candidate | 2017-2021 | No | [Croatia NAP](https://zdravstvo.gov.hr/programi-i-projekti/nacionalni-programi-projekti-i-strategije/ostali-programi/nacionalni-program-za-kontrolu-otpornosti-bakterija-na-antibiotike-2017-2021/2198) |
| Czechia | Member | 2019-2022 | No | [Czechia NAP](https://www.svscr.cz/wp-content/files/zvirata/AP_NAP_2019_-_text.pdf) |
| Denmark | Member | 2021-2023 | Yes | [Denmark NAP](https://foedevarestyrelsen.dk/Media/638225168289023378/The%20Danish%20Veterinary%20and%20Food%20Administrations%20national%20action%20plan%20for%20AMR%20in%20production%20animals%20and%20food_2021-2023.pdf) |
| Estonia | Member | 2021-2026 | Yes | [Estonia NAP](https://www.agri.ee/sites/default/files/documents/2021-09/tegevuskava-amr-2021-2026.pdf) |
| Finland | Member | 2017-2021 | Yes | [Finland NAP](https://stm.fi/documents/1271139/1359637/12_17_National_Action_Plan_on_Antimicrobial_Resistance_2017_2021_V1+(002).pdf/9ff43364-6cc2-4e39-b0cf-5664460a3d9e/12_17_National_Action_Plan_on_Antimicrobial_Resistance_2017_2021_V1+(002).pdf?t=1535980865000) |
| France | Member | 2023-2028 | Yes | [France NAP](https://agriculture.gouv.fr/le-plan-ecoantibio-3-2023-2028) |
| Germany | Member | 2024-2026 | Yes | [Germany NAP](https://www.bundesgesundheitsministerium.de/fileadmin/Dateien/3_Downloads/A/Antibiotika-Resistenz-Strategie/240510_1._Aktionsplan_zur_DART_2030.pdf) |
| Greece | Member | 2019-2023 | Yes | [Greece NAP](https://www.minagric.gr/for-citizen-2/ktiniatrikafarmakeytika-politis/9889-ethniko-sxedio-drash2019-2023) |
| Hungary | Member | 2019 (published) | Yes | [Hungary NAP](http://info.nevesforum.hu/wp-content/uploads/2021/03/AMR_strategia_rovid.pdf) |
| Iceland | Member | 2025-2029 | No | [Iceland NAP](https://www.stjornarradid.is/library/04-Raduneytin/Heilbrigdisraduneytid/ymsar-skrar/A%c3%b0ger%c3%b0a%c3%a1%c3%a6tlun%20gegn%20s%c3%bdklalyfja%c3%b3n%c3%a6mi.pdf) |
| India | Key partner | 2017-2021 | No | [India NAP](https://ncdc.mohfw.gov.in/wp-content/uploads/2024/03/File645.pdf) |
| Indonesia | Key partner/Candidate | 2020-2024 | Yes | [Indonesia NAP](https://uapt33090-my.sharepoint.com/personal/jacinta_pinho_ua_pt/Documents/Artigo%20Guidelines%20Veterinária/Indonesia%20NAP) |
| Ireland | Member | 2021-2025 | Yes | [Ireland NAP](https://cdn.who.int/media/docs/default-source/antimicrobial-resistance/amr-spc-npm/nap-library/ireland_nap_2.0.pdf?sfvrsn=744ee5d_3&download=true) |
| Israel | Member | (under development) | No | [Israel NAP](https://pubmed.ncbi.nlm.nih.gov/37101188/) |
| Italy | Member | 2022-2025 | Yes | [Italy NAP](https://www.salute.gov.it/imgs/C_17_pubblicazioni_3294_allegato.pdf) |
| Japan | Member | 2023-2027 | Yes | [Japan NAP](https://www.mhlw.go.jp/content/10900000/001096228.pdf) |
| Republic of Korea | Member | 2021-2025 | Yes | [Republic of Korea NAP](https://www.mohw.go.kr/boardDownload.es?bid=0027&list_no=368388&seq=4) |
| Latvia | Member | 2019-2020 | Yes | [Latvia NAP](https://faolex.fao.org/docs/pdf/lat196392.pdf) |
| Lithuania | Member | 2017-2021 | No | [Lithuania NAP](https://faolex.fao.org/docs/pdf/lit202520.pdf) |
| Luxemburg | Member | 2018-2022 (extended until 2024) | Yes | [Luxemburg NAP](https://sante.public.lu/fr/publications/p/plan-national-antibiotiques-2018-2022.html) |
| Mexico | Member | 2023-2024 | Yes | [Mexico NAP](https://www.gob.mx/cms/uploads/attachment/file/847156/Plan_Estrategico_RAM_VF.pdf) |
| Netherlands | Member | 2024-2030 | Yes | [The Netherlands NAP](https://open.overheid.nl/documenten/686e0ef0-5074-4fac-aa57-f5a69d59dc7a/file) |
| New Zealand | Member | 2017 (published); New plan is under development. | Yes | [New Zealand NAP](https://www.health.govt.nz/system/files/documents/publications/new-zealand-antimicrobial-resistance-action-plan.pdf) |
| Norway | Member | 2015-2020 | Yes | [Norway NAP](https://www.regjeringen.no/contentassets/5eaf66ac392143b3b2054aed90b85210/antibiotic-resistance-engelsk-lavopploslig-versjon-for-nett-10-09-15.pdf) |
| Peru | Candidate | 2019-2021 | No | [Peru NAP](https://cdn.www.gob.pe/uploads/document/file/2399569/Plan%20Multisectorial%20para%20enfrentar%20la%20Resistencia%20a%20los%20Antimicrobianos%202019%20-%202021.pdf.pdf?v=1636729188) |
| Poland | Member | To be applied on June 14, 2024 | Yes | [Poland NAP](https://www.gov.pl/attachment/2cef1578-67d3-46f1-a45c-db4e689d8e67) |
| Portugal | Member | 2019-2023 (new plan under development) | Yes | [Portugal NAP](https://faolex.fao.org/docs/pdf/por221831.pdf) |
| Romania | Candidate | 2023-2030 | Yes | [Romania NAP](https://legislatie.just.ro/Public/DetaliiDocument/275713) |
| Slovakia | Member | 2019-2021 | No | [Slovakia NAP](https://www.svps.sk/zdroje/data/10/Plan_AMR_2019-2021_EN.pdf) |
| Slovenia | Member | 2023-2027 | Yes | [Slovenia NAP](https://skp.si/wp-content/uploads/2021/12/Predlog_SN_SKP_22.12.2021_koncna_cista.pdf) |
| South Africa | Key partner | 2017-2024 | Yes | [South Africa NAP](https://knowledgehub.health.gov.za/system/files/elibdownloads/2020-03/AMR%20National%20Action%20Plan%202018%20-%202024.pdf) |
| Spain | Member | 2022-2024 | Yes | [Spain NAP](https://www.resistenciaantibioticos.es/es/publicaciones/plan-nacional-frente-la-resistencia-los-antibioticos-pran-2022-2024) |
| Sweden | Member | 2024-2025 | Yes | [Sweden NAP](https://www.government.se/contentassets/1fedc516373d421f919814f1963e2fe1/amr_strategi_eng_web_ny2.pdf) |
| Switzerland | Member | 2015 (published) | Yes | [Switzerland NAP](https://www.star.admin.ch/star/fr/home/strategiestar/landingstar.html) |
| Thailand | Candidate | 2023-2027 | Yes | [Thailand NAP](https://plan.fda.moph.go.th/media.php?id=532732166124216320&name=binder18894.pdf) |
| Türkiye | Member | 2019-2023 | No | [Turkey NAP](https://faolex.fao.org/docs/pdf/tur193517.pdf) |
| United Kingdom | Member | 2024-2029 | Yes | [United Kingdom NAP](https://www.gov.uk/government/publications/uk-5-year-action-plan-for-antimicrobial-resistance-2024-to-2029/confronting-antimicrobial-resistance-2024-to-2029) |
| United States | Member | 2020-2025 | Yes | [United States NAP](https://aspe.hhs.gov/sites/default/files/migrated_legacy_files/196436/CARB-National-Action-Plan-2020-2025.pdf) |

# **Appendix Table S5: Lists of medically important antimicrobials (MIAs) in each guideline (n=82).**

| Country | Antimicrobial Agents | Observations | Guidelines |
| --- | --- | --- | --- |
| Australia | Low importance: neomycin*, framycetin*, streptomycin*, dihydrostreptomycin^£,^*, paromomycin^ǂ,^***, chloramphenicol***, florfenicol, azithromycin*^,^***, clarithromycin***, erythromycin*, roxithromycin***, spiramycin***, oleandomycin^£^, tilmicosin, tulathromycin, tylosin, amoxicillin*, ampicillin*, benzylpenicillin*, phenoxymethylpenicillin*, procaine penicillin^ǂ^, benzathine penicillin^ǂ^, penethamate hydriodide*^,£^, piperacillin^£^, tiamulin, retapamulin***, lefamulin^ǂ^, bacitracin, gramicidin^ǂ,^***, sulfadiazine, trimethoprim, proguanil^ǂ^, pyrimethamine***, sulfadimidine^£^, sulfaquinoxaline^ǂ,£^, sulfamerazine^£,^***, sulfathiazole^£,^***, phthalylsulfathiazole^£,^***, tetracycline***, doxycycline***, minocycline***, chlortetracycline^£^, oxytetracycline^£^, sulfamethoxypyridazine, sulfametomidine  Medium Importance: amoxicillin + clavulanic acid, gentamicin, tobramycin, spectinomycin, apramycin^£^, cefalexin***, cefalotin***, cefazolin***, cefapirin^£^, cefalonium, cefaclor***, cefuroxime, ceftriaxone***, cefotaxime***, ceftiofur, cefovecin, ceftazidime***, cefepime***, ceftaroline, cefoxitin***, clindamycin***, lincomycin, daptomycin***, metronizadole***, tinidazole***, flucloxacillin***, dicloxacillin***, cloxacillin^£^, mupirocin^£^, sulfamethoxazole + trimethoprim, sulfadoxine + trimethoprim^£^, sulfadiazine + trimethoprim^£^, sulfadimidine + trimethoprim^£^  High Importance: piperacillin + tazobactam, ticarcillin + clavulanic acid, ceftolozane + tazobactam^ǂ^, amikacin, clofazimine, dapsone, isoniazid, ethambutol, pyrazinamide, cycloserine, *para*-aminosalicylic acid, protionamide, capreomycin, imipenem, meropenem, ertapenem, fosfomycin***, fusidic acid***, vancomycin***, teicoplanin***, norvancomycin^ǂ^, tigecycline***, aztreonam, nitrofurantoin***, furazolidone***, linezolid***, tedizolid^$^, polymyxin B, colistin***, norfloxacin***, ciprofloxacin***, moxifloxacin***, ofloxacin***, levofloxacin***, enrofloxacin***, enrofloxacin + silver^ǂ,^***, sulfadiazine***, ibafloxacin***, marbofloxacin***, orbifloxacin***, pradofloxacin***, rifampicin***, rifabutin***, rifaximin***, quinupristin + dalfopristin***, pristinamycin***, virginiamycin*** | Australian Strategic and Technical Advisory Group on Antimicrobial Resistance (ASTAG) list (2018)[82] | [1-4] |
| Austria | All | WHO (2019)[83] and WOAH (2021)[84] | [5] |
| Belgium | Yellow: cefalexin, chloramphenicol, florfenicol, thiamphenicol, benzylpenicillin (= penicillin G), cloxacillin, nafcillin, penethamate, phenoxymethylpenicillin (= penicillin V), tiamulin, valnemulin, trimethoprim, sulfadiazine, sulfadimethoxine, sulfadoxineǂ, sulfamides + trimethoprim  Orange: amikacin, apramycin, dihydrostreptomycin, framycetin, gentamicin, kanamycin, neomycin, paromomycin, spectinomycin, clindamycin, lincomycin, pirlimycin, erythromycin, gamithromycin, spiramycin, tildipirosin, tilmicosin, tulathromycin, tylosin, tylvalosin, amoxicillin, ampicillin, polymyxin B, colistin, chlortetracycline, doxycycline, oxytetracycline, tetracycline, amoxicillin + clavulanic acid, cloxacillin + ampicillinǂ, lincomycin + spectinomycinǂ, lincomycin + neomycinǂ, macrolide + rifamycinǂ, metronidazole + spiramycinǂ, neomycin + polymyxin Bǂ, penethamate + benethamineǂ, penicillin + framycetinǂ, procaine benzylpenicillin + neomycinǂ, penicillin + aminoglycosideǂ, penicillin G + neomycinǂ, procaine benzylpenicillin + dihydrostreptomycinǂ, procaine benzylpenicillin + nafcillinǂ, procaine benzylpenicillin + neomycinǂ, aminobenzylpenicillin + aminoglycosideǂ, cefalexin + kanamycinǂ  Red: cefovecin, cefquinome, ceftiofur, danofloxacin, difloxacin, enrofloxacin, flumequine, marbofloxacin, orbifloxacin, pradofloxacin, cefquinome + aminoglycosideǂ | AMCRA Vade-mecum (https://formularium.amcra.be/), based on AMEG (2019)[85] | [6-8] |
| Brazil | All | WHO (2019)[83] and WOAH (2021)[84] | [9] |
| Bulgaria | AMEG | AMEG (2019)[85] | [10] |
| Canada | Carbapenems, cephalosporins 3^rd^ and 4^th^ generations, (fluoro)quinolones, glycopeptides**, glycylcyclines**, ketolides**, lipopeptides**, monobactams**, nitroimidazoles, oxazolidinones**, penicillins, streptogramins, ethambutol**, isoniazid**, pyrazinamide**, rifampicin, aminoglycosides, cephalosporins 1^st^ and 2^nd^ generations, fusidic acid, lincosamides, macrolides, trimethoprim + sulfamethoxazole, aminocyclitols, bacitracins, fosfomycin, nitrofurans, phenicols, sulfonamides, tetracyclines, trimethoprim | Veterinary Drug Directorate’s (VDD) Categorization of Antimicrobial Drugs Based on their Importance in Human Medicine (2009)[86] | [11] |
| Colombia | All | WHO (2019)[83] and WOAH (2019)[87] | [12] |
|  | n.a. | n.a. | [13] |
| Costa Rica | n.a. | n.a. | [14] |
| Denmark | n.a. | n.a. | [88] |
|  | Florfenicol, lincomycin, macrolides (e.g. tylosin, tylvalosin, tilmicosin, tildipirosin, tulathromycin, gamithromycin), neomycin, penicillins narrow-spectrum (e.g. benzylpenicillin, benethaminpenicillin, phenoxymethylpenicillin), penicillins broad-spectrum, (e.g. amoxycillin, ampicillin possibly with clavulanic acid), pleuromutilins (e.g. tiamulin, valnemulin), sulfonamides (e.g. sulfadizine, sulfadimidine, sulfadoxineǂ possibly in combination with trimethoprim), spectinomycin, streptomycin, apramycin, gentamicin, tetracycline (e.g. chlortetracycline, oxytetracycline, doxycycline), 3^rd^ and 4^th^ generation cephalosporins (e.g. ceftiofur, cefquinome), colistin, fluoroquinolones (e.g. enrofloxacin) | The Danish Veterinary and Food  Administration Recommendations | [15] |
| Estonia | All | WHO (no date) | [16] |
| Finland | n.a. | n.a. | [17] |
| France | n.a. | n.a. | [18] |
| Germany | n.a. | n.a. | [19] |
| Greece | n.a. | n.a. | [20] |
| Hungary | n.a. | n.a. | [21] |
| Indonesia | All | Mentions WHO (2016)[89], AMEG (no date), WOAH (2015)[90] | [22] |
|  | All | Mentions WHO (2017)[91] and WOAH (2019)[83] | [23] |
| Ireland | AMEG | AMEG (no date)[92] | [24] |
|  | n.a. | n.a. | [25] |
|  | n.a. | n.a. | [26] |
|  | n.a. | n.a. | [27] |
|  | n.a. | n.a. | [28] |
|  | AMEG | AMEG (no date)[92] | [29] |
| Italy | All | Mentions WHO (no date) and WOAH (no date) | [30] |
|  | All | WHO (no date) and WOAH (2015)[93] | [31] |
|  | All | WHO (2019)[83] | [32] |
|  | All | WHO (2019)[83] and AMEG (2019)[85] | [33] |
| Japan | Enrofloxacin, ofloxacin, orbifloxacin, danofloxacin, norfloxacin, marbofloxacin, lomefloxacin, pradofloxacin, cefovecin, cefquinome, ceftiofur, cefpodoxime, tulathromycin, gamithromycin, colistin | Veterinary Drug Inspection Laboratory of the Ministry of Agriculture | [34] |
|  | Rank III: macrolides with 16-membered ring, astromicin, framycetin, kanamycin, quinolones*, sulfonamides, natural tetracyclines, 1^st^ generation cephalosporins, nitroimidazoles, fusidic acid, polypeptides  Rank II: products with β-lactamase inhibitors, gentamicin, sisomicin, streptomycin, chloramphenicol, streptogramins, spectinomycin, sulfamethoxazole + trimethoprim, tetracyclines, penicillins, penem-based, fosfomycin, lincomycin, erythromycin  Rank I: Macrolides with 14- and 15-membered ring structures (except erythromycin), oxazolidinones, kanamycin, arbekacin, carbapenems, glycopeptides, glycylcyclines, antituberculosis drugs, 3^rd^ and 4^th^ generation cephalosporins, polypeptides, colistin, polymyxin B, fluoroquinolones, mupirocin, monobactams, lipopeptides | National list - Food Safety Commission  Ranking^(a)^ | [35] |
| Korea (Republic of Korea) | Erythromycin, spiramycin, tildipirosin, tilmicosin, tylosin, tulathromycin, kitasamycin, oleandomycin, roxithromycin, sulfachlorpyridazine, sulfadoxine^ǂ^, sulfaguanidine, sulfamethazine, sulfamonomethoxine^ǂ^, sulfaquinoxaline, sulfaclozine^ǂ^, sulfisomidine, sulfadiazine, sulfadimethoxine, sulfamerazine, sulfamethoxypyridazine, sulfanilamide, sulfamethoxazole, sulfathiazole, sulfisoxazole, trimethoprim, bacitracin, colistin, enramycin, virginiamycin, tiamulin, valnemulin, amoxicillin, ampicillin, cloxacillin, cefalexin, cefazolin, ceftiofur, cefquinome, nalidixic acid, flumequine, oxolinic acid, danofloxacin, enrofloxacin, marbofloxacin, apramycin, spectinomycin, amikacin, streptomycin, gentamicin, kanamycin, neomycin, doxycycline, tetracycline, minocycline, clindamycin, lincomycin, chloramphenicol, thiamphenicol, florfenicol | National list based on WHO (2019)[83] and WOAH (2018)[94] | [36-38] |
| Latvia | All | WHO (2017)[91] and WOAH (2015)[90] | [39] |
| Luxembourg | Yellow: cefalexin, chloramphenicol, florfenicol, thiamphenicol, benzylpenicillin, cloxacillin, nafcillin, penethamate, phenoxymethylpenicillin, tiamulin, valnemulin, trimethoprim, sulfadiazine, sulfadimethoxine, sulfadoxineǂ, sulfamides + trimethoprim  Orange: amikacin, apramycin, dihydrostreptomycin, framycetin, gentamicin, kanamycin, neomycin, paromomycin, spectinomycin, clindamycin, lincomycin, pirlimycin, erythromycin, gamithromycin, spiramycin, tildipirosin, tilmicosin, tulathromycin, tylosin, tylvalosin, amoxicillin, ampicillin, polymyxin B, colistin, chlortetracycline, doxycycline, oxytetracycline, tetracycline, amoxicillin + clavulanic acid, cloxacillin + ampicillinǂ, lincomycin + spectinomycinǂ, lincomycin + neomycinǂ, macrolide + rifamycinǂ, metronidazole + spiramycinǂ, neomycin + polymyxin Bǂ, penethamate + benethamineǂ, penicillin + framycetinǂ, procaine benzylpenicillin + neomycinǂ, penicillin + aminoglycosideǂ, penicillin G + neomycinǂ, procaine benzylpenicillin + dihydrostreptomycinǂ, procaine benzylpenicillin + nafcillinǂ, procaine benzylpenicillin + neomycinǂ, aminobenzylpenicillin + aminoglycosideǂ, cefalexin + kanamycinǂ  Red: cefovecin, cefquinome, ceftiofur, danofloxacin, difloxacin, enrofloxacin, flumequine, marbofloxacin, orbifloxacin, pradofloxacin, cefquinome + aminoglycosideǂ | AMCRA Vade-mecum (https://formularium.amcra.be/), based on AMEG (2019)[85] | [40] |
| Mexico | n.a. | n.a. | [41] |
|  | Amoxicillin, doxycycline, oxytetracycline, chlortetracycline, dicloxacillin, benzylpenicillin, benzathine + benzylpenicillin, procaine benzylpenicillin, sulfadiazine, sulfadoxineǂ, sulfamethoxazole, sulfamerazine, trimethoprim, bacitracin, tilmicosin, tylosin, tylvalosin, tulathromycin, streptomycin, gentamicin, dihydrostreptomycin, kanamycin, neomycin, florfenicol, cefalexin, cefquinome, clindamycin, lincomycin, enrofloxacin, norfloxacin, polymyxin B, colistin, ceftiofur, fosfomycin | National list | [42] |
| Netherlands | First choice: cloxacillin, nafcillin, phenoxymethylpenicillin (penicillin V), benzylpenicillin (penicillin G), penethamate hydroiodide, chlortetracycline, doxycycline, oxytetracycline, tetracycline, florfenicol, sulfachlorpyridazine, sulfadiazine, sulfadimethoxine, sulfadimidine, sulfadoxineǂ, sulfamethoxazole, sulfaquinoxaline, trimethoprim, lincomycin, pirlimycin, erythromycin*, tylosin*, spiramycin*, tylvalosin*, tilmicosin*, tiamulin, valnemulin, bacitracin, trimethoprim + sulfonamides  Second choice: amoxicillin, ampicillin, amoxicillin + clavulanic acid, cefalexin, cefalonium, cefapirin, gamithromycin, tulathromycin, tildipirosin, apramycin, dihydrostreptomycin, framycetin, gentamicin, kanamycin, neomycin, paromomycin, spectinomycin, flumequine*, oxolinic acid*, colistin*, penicillin + kanamycinǂ, penicillin + neomycinǂ, penicillin + dihydrostreptomycinǂ, amoxicillin + colistinǂ, cefalexin + kanamycinǂ, lincomycin + spectinomycinǂ  Third choice: cefoperazone, cefquinome, ceftiofur, danofloxacin, difloxacin, enrofloxacin, marbofloxacin  Forbidden for food-producing animals: cefadroxil***, cefovecin***, chloramphenicol***, clindamycin***, orbifloxacin***, pradofloxacin***, polymyxin B***, fusidic acid***, metronidazole***, metronidazole + spiramycinǂ | National classification^(c)^  (The Veterinary Antimicrobial Policy Working Group – WVAB)[95] | [43-49] |
| New Zealand | Green: procaine penicillin, penethamate hydroiodide, tetracyclines  Orange: aminoglycosides, ampicillin, clavulanic acid, cloxacillin, 1^st^ and 2^nd^ generation cephalosporins, lincosamides, potentiated sulfonamides  Red: 3^rd^ and 4^th^ generation cephalosporins, fluoroquinolones, macrolides | National classification^(d)^ based on WHO (no date) and WOAH (no date) | [50-53] |
| Norway | AMEG | AMEG (2019)[85] | [54] |
| Poland | 3^rd^ and 4^th^ generation cephalosporins, fluoroquinolones, phenicols, glycopeptides, carbapenems**, piperacillin**, piperacillin + tazobactam** | n.a. | [55] |
| Portugal | n.a. | n.a. | [56] |
|  | AMEG | AMEG (2019)[85] | [57] |
| Romania | AMEG | AMEG (2019)[85] | [58] |
| Slovenia | All | WHO (no date) and WOAH (no date) | [59] |
| South Africa | n.a. | n.a. | [60] |
|  | All | WHO (2011)[96] and WOAH (2015)[90] | [61] |
| Spain | n.a. | n.a. | [62] |
|  | n.a. | n.a. | [63] |
|  | n.a. | n.a. | [64] |
|  | Category A (Avoid): mecillinam, pivmecillinam, telithromycin**, aztreonam**, rifampicin, piperacillin + tazobactam**, meropenem**, doripenem**, daptomycin**, linezolid**, clofazimine**, dapsone**, pristinamycin, virginiamycin, isoniazid**, ethambutol**, pyrazinamide**, ethionamide**, ceftobiprole**, ceftaroline**, ceftolozane + tazobactam**, faropenem**, vancomycin**, tigecycline**, fosfomycin, mupirocin**  Category B (Restricted): cefoperazone, cefquinome, ceftiofur, danofloxacin, enrofloxacin, marbofloxacin, flumequine, pradofloxacin, orbifloxacin, colistin  Category C (Caution): apramycin, framycetin, dihydrostreptomycin, gentamicin, kanamycin, neomycin, paromomycin, amoxicillin/clavulanic acid, florfenicol, thiamphenicol, cefacetrile, cefalexin, cefalonium, cefapirin, erythromycin, gamithromycin, spiramycin, tildipirosin, tilmicosin, tulathromycin, tylosin, lincomycin, pirlimycin, rifaximin  Category D (Prudence): spectinomycin, amoxicillin, ampicillin, cloxacillin, benzylpenicillin, penethamate hydriodide, sulfadiazine, sulfadimidine, sulfadimethoxine, sulfadoxineǂ, trimethoprim, chlortetracycline, doxycycline, oxytetracycline, tetracycline | Based on AMEG (no date; table on the guideline itself) | [65] |
|  | Category A (Avoid): ceftobiprole**, ceftaroline**, meropenem**, doripenem**, fosfomycin, vancomycin**, tigecycline**, daptomycin**, aztreonam**, linezolid**, clofazimine**, dapsone**, isoniazid**, ethambutol**, pyrazinamide**, ethionamide**  Category B (Restricted): cefoperazone, cefovecin, cefquinome, ceftiofur, danofloxacin, enrofloxacin, marbofloxacin, pradofloxacin, orbifloxacin, flumequine, colistin, polymyxin B  Category C (Caution): apramycin, framycetin, dihydrostreptomycin, gentamicin, kanamycin, neomycin, paromomycin, amoxicillin + clavulanic acid, chloramphenicol, florfenicol, thiamphenicol, cefacetrile, cefadroxil, cefalexin, cefalonium, cefalotin, cefapirin, cefazolin, erythromycin, gamithromycin, spiramycin, tildipirosin, tilmicosin, tulathromycin, tylosin, tylvalosin, clindamycin, lincomycin, pirlimycin, tiamulin  valnemulin, rifaximin  Category D (Prudence): bacitracin, spectinomycin, metronidazole, amoxicillin, ampicillin, metampicillin, cloxacillin, benzylpenicillin, phenoxymethylpenicillin, penethamate hydriodide, fusidic acid, sulfadiazine, sulfadimidine, sulfamethoxazole, sulfamethoxypyridazine, sulfadimethoxine, sulfaquinoxaline, sulfadoxineǂ, trimethoprim, chlortetracycline, doxycycline, oxytetracycline, tetracycline, lincomycin, clindamycin, tiamulin, valnemulin, bacitracin, colistin, polymyxin, thiostreptonǂ, fusidic acid | Based on AMEG (no date; external link - <https://www.resistenciaantibioticos.es/es/lineas-de-accion/vigilancia/antibioticos-criticos>) | [66] |
| Sweden | n.a. | n.a. | [67] |
|  | Fluoroquinolones, cephalosporins and macrolides | Refers to WHO classification (no date) | [68, 69] |
| Switzerland | All | Refers to WHO (2012)[97] and WOAH (no date) classifications | [70] |
|  | All | Refers to WHO (2012)[97] and WOAH (no date) classifications | [71] |
| Thailand | n.a. | n.a. | [72] |
|  | All | Mentions WHO (2016)[89], AMEG (no date), WOAH (2015)[90] | [22] |
| United Kingdom | AMEG category A (forbidden): ceftobiprole**, ceftaroline**, meropenem**, doripenem**, fosfomycin, vancomycin**, tigecycline**, daptomycin**, aztreonam**, linezolid**, clofazimine**, dapsone**, isoniazid**, ethambutol**, pyrazinamide**, ethionamide**  Class Three: Enrofloxacin, marbofloxacin, ceftiofur, cefquinome, colistin  Class Two: Amoxicillin + clavulanic acid, apramicyn, neomycin, paromomycin, streptomycin, tiamulin, valnemulin, florfenicol, lincomycin, tylosin, tyvalosin, tilmicosin, tulathromycin, tildipirosin  Class One: Trimethoprim, chlortetracycline, doxycycline, oxytetracycline, tetracycline, phenoxymethylpenicillin, procaine benzylpenicillin, ampicillin, spectinomycin | The Pig Veterinary Society (2020) (Based on AMEG)^(e)^  (external link: <https://www.pigvetsoc.org.uk/files/document/558/2004%20PVS%20AntiB%20Prescribing%20Policy.pdf>) | [74] |
|  | AMEG Category B/HPCIAs: cefoperazone, cefovecin, cefquinome, ceftiofur, colistin, polymyxin B, cinoxacin, danofloxacin, difloxacin, enrofloxacin, flumequine, ibafloxacin, marbofloxacin, norfloxacin, orbifloxacin, oxolinic acid, pradofloxacin | List based on AMEG (Category B/HPCIAs) | [74] |
|  | AMEG Category B/HPCIAs: cefoperazone, cefovecin, cefquinome, ceftiofur, colistin, polymyxin B, cinoxacin, danofloxacin, difloxacin, enrofloxacin, flumequine, ibafloxacin, marbofloxacin, norfloxacin, orbifloxacin, oxolinic acid, pradofloxacin | List designated by Veterinary Medicine Directorate (VMD) and based on AMEG (Category B/HPCIAs) | [75] |
|  | Colistin, fluoroquinolones and 3^rd^ and 4^th^ generation cephalosporins | n.a. | [76] |
|  | AMEG | AMEG (2020)[98] | [77-79] |
| United States | Benzylpenicillin, phenoxymethylpenicillin, cloxacillin, dicloxacillin, methicillin***, oxacillin, amoxicillin, ampicillin, bacampicillin***, hetacillin, carbenicillin, mezlocillin, piperacillin, ticarcillin**, amoxicillin + clavulanic acid*** ampicillin + sulbactam***, piperacillin + tazobactam, ceftolozane + tazobactam, ceftazidime + avibactam, cefuroxime***, cefprozil***, loracarbef***, cefoxitin***, cefotetan***, cefotiam***, cefmetazole***, cefaclor***, ceforanide***, cefamandole***, cefonicid***, cephalexin, cefadroxil, cefazolin***, cefdinir***, cefditoren***, cefepime***, cefixime***, cefmenoxime***, cefoperazone***, cefotaxime***, cefovecin, cefpiramide***, cefpodoxime, ceftazidime***, ceftibuten***, ceftiofur***, ceftizoxime***, ceftolozane***, ceftriaxone***, amikacin, apramycin, arbekacin***, astromicin***, bekanamycin***, dibekacin***, gentamicin, kanamycin, neomycin, netilmicin***, paromomycin***, streptomycin, tobramycin***, bedaquiline***, capreomycin***, cycloserine***, ethambutol, ethionamide***, isoniazid***, *para*-aminosalicylic acid***, pretomanid***, pyrazinamide***, sodium aminosalicylate***, doripenem, ertapenem, imipenem, meropenem, imipenem + cilastatin, imipenem + relebactam, meropenem + vaborbactam, besifloxacin***, cinoxacin***, ciprofloxacin, danofloxacin, delafloxacin***, difloxacin, enoxacin***, enrofloxacin***, gatifloxacin***, gemifloxacin***, grepafloxacin***, levofloxacin***, lomefloxacin***, marbofloxacin, moxifloxacin***, nalidixic acid***, norfloxacin***, ofloxacin***, orbifloxacin, ozenoxacin***, pradofloxacin***, sparfloxacin***, trovafloxacin***, fosfomycin***, dalbavancin, oritavancin, telavancin, vancomycin, daptomycin, fidaxomicin, azithromycin, clarithromycin, dirithromycin***, erythromycin, gamithromycin, oleandomycin***, tildipirosin, tilmicosin, troleandomycin, tulathromycin, tylosin, tylvalosin, methenamine***, aztreonam, furazolidone***, nifuroxazide***, nitrofurantoin***, metronidazole***, secnidazole***, tinidazole***, benznidazole^ǂ^, linezolid, tedizolid, chloramphenicol***, florfenicol, lefamulin, retapamulin***, tiamulin, colistin, polymyxin B, rifabutin***, rifamycin***, rifapentine***, rifaximin***, quinupristin + dalfopristin***, virginiamycin, chlortetracycline, demeclocycline***, doxycycline***, minocycline***, oxytetracycline, sarecycline***, tetracycline, ormetoprim, pyrimethamine***, sulfachlorpyridazine, sulfadiazine, sulfadimethoxine, sulfafurazole, sulfamerazine, sulfamethazine, sulfamethizole, sulfamethoxazole***, sulfanilamide, sulfaphenazole***, sulfapyridine***, sulfaquinoxaline, sulfathiazole***, trimethoprim, bacitracin | FDA GFI #152 (external links for complete information)  <https://animaldrugsatfda.fda.gov/adafda/views/#/search>  <https://www.accessdata.fda.gov/scripts/cder/ob/index.cfm> | [80, 81] |

* An antimicrobial agent that is categorized as HPCIA or CIA but is considered in the lowest rating for other national lists

** Authorized for use in humans only (WHO)

£ Not used in humans (ASTAG)

*** Not authorized for use in food-producing animals (ASTAG, WVAB, FDA)

ǂ Not listed by WHO as MIAs

(a) Rank I: very high importance; Rank II: highly important; Rank III: Important.

(b) Group 1: first line antimicrobials; Group 2: alternative antimicrobials when first line antimicrobials are not effective.

(c) First choice: Empirical therapy with antimicrobial agents that are effective against the indication and do not have a specific negative resistance inducing effect according to current insights; second choice: No, unless substantiated by sensitivity tests and/or company history regarding occurrence of resistance in animal pathogens; third choice: These are critical antimicrobials to human health care. No, unless for individual animals and based on bacteriological tests showing that there are no alternatives.

(d) Green: first line under therapeutic conditions; orange: restricted to specific situations or used as second-line therapy; red: important in treating refractory conditions in human and veterinary medicine. Only use after diagnosis and based on sufficient evidence to justify need.

(e) Class One (EMA Category D, Use with prudence): first line treatment choice whenever possible, prudently, and only when clinically required; Class Two (EMA Category C, Use with caution): choose only when there are no clinically effective alternatives in Category D, supported by sensitivity testing; Class Three (EMA Category B, Restrict use): antibiotics in this category are critically

important in human medicine and use in animals should be restricted to mitigate the risk to public health, being considered as last resort products, to consider only when no antibiotics in category C or D could be clinically effective, supported by sensitivity testing.

n.a.: not available

Note: The WHO MIAs list was first developed in 2005[99] and updated in 2007,[100] 2011,[96] 2012,[97] 2016,[89] 2017,[91] 2019,[83] and 2024.[101]

# **Appendix Table S6: AGREE II standardized scores by domain (D) for the 42 clinical guidelines considered in the quality appraisal.**

| Country | Guideline | D1: Scope and purpose (%) | D2: Stakeholder involvement (%) | D3: Rigor of development (%) | D4: Clarity of presentation (%) | D5: Applicability (%) | D6: Editorial independence (%) | Total Score | Overall score (%) |
| --- | --- | --- | --- | --- | --- | --- | --- | --- | --- |
| Australia | Antimicrobial prescribing guidelines for sheep [2] | 94·44 | 88·89 | 48·96 | 91·67 | 22·92 | 37·50 | 106·0 | 64·1 |
|  | Antimicrobial prescribing guidelines for dairy cattle [3] | 97·22 | 88·89 | 48·96 | 91·67 | 20·83 | 37·50 | 106·0 | 64·2 |
|  | Antimicrobial prescribing guidelines for feedlot cattle [102] | 91·67 | 75·00 | 47·92 | 75·00 | 22·92 | 50·00 | 101·0 | 60·4 |
|  | Antimicrobial prescribing guidelines for pigs [1] | 94·44 | 88·89 | 46·88 | 91·67 | 22·92 | 37·50 | 105·0 | 63·7 |
| Austria | Leitlinien für den sorgfältigen umgang mit antibakteriell wirksamen tierarzneimitteln [103] | 61·11 | 16·67 | 9·38 | 44·44 | 10·42 | 4·17 | 52·5 | 24·4 |
| Belgium | Surveillance et utilisation des antibiotiques, y compris de ceux d’importance critique, chez les chevaux [104] | 47·22 | 33·33 | 9·38 | 61·11 | 12·50 | 4·17 | 56·5 | 28·0 |
| Canada | Antimicrobial prudent use guidelines for beef cattle, dairy cattle, poultry, and swine [105] | 66·67 | 47·22 | 17·71 | 91·67 | 6·25 | 25·00 | 73·0 | 42·4 |
| Denmark | Guideline for prescribing antimicrobial for pigs [106] | 69·44 | 33·33 | 12·50 | 47·22 | 4·17 | 4·17 | 57·5 | 28·5 |
| Estonia | Juhend antibiootikumide kasutamiseks põllumajandusloomadel [107] | 52·78 | 19·44 | 9·38 | 86·11 | 14·58 | 0·00 | 59·5 | 30·4 |
| Finland | Recommendations for the use of antimicrobials in the treatment of the most significant infectious and contagious diseases in animals [108] | 80·56 | 44·44 | 18·75 | 83·33 | 4·17 | 4·17 | 71·0 | 39·2 |
| Germany | Guidelines for the prudent use of veterinary antimicrobial drugs -with notes for guidance- [109] | 61·11 | 47·22 | 23·96 | 77·78 | 8·33 | 4·17 | 70·5 | 37·1 |
| Hungary | Az antibiotikum - kezelés minimumkövetelményeire vonatkozó útmutató [110] | 30·56 | 8·33 | 3·13 | 16·67 | 2·08 | 4·17 | 35·5 | 10·8 |
| Korea | 산업동물 수의사를 위한 항생제 길잡이 [111] | 47·22 | 44·44 | 18·75 | 72·22 | 14·58 | 4·17 | 65·5 | 33·6 |
|  | 소 항생제 처방 가이드라인 [112] | 36·11 | 27·78 | 7·29 | 61·11 | 12·50 | 4·17 | 52·5 | 24·8 |
|  | 돼지 항생제 처방 가이드라인 [113] | 36·11 | 27·78 | 7·29 | 72·22 | 12·50 | 4·17 | 54·5 | 26·7 |
| Luxemburg | Empfehlungen für den sachgemäßen einsatz von antibiotika in der tiergesundheit [114] | 52·78 | 44·44 | 27·08 | 50·00 | 6·25 | 4·17 | 64·5 | 30·8 |
| Netherlands | Werkgroep vterinair antibioticumbeleid - formularium melkvee [115] | 77·78 | 41·67 | 14·58 | 63·89 | 14·58 | 20·83 | 69·0 | 38·9 |
|  | Werkgroep vterinair antibioticumbeleid - formularium paard [116] | 77·78 | 41·67 | 14·58 | 63·89 | 14·58 | 20·83 | 69·0 | 38·9 |
|  | Formularium vleeskalveren en vleesvee [117] | 80·56 | 41·67 | 14·58 | 63·89 | 6·25 | 20·83 | 67·5 | 38·0 |
|  | Formularium kleine herkauwers – schaap [118] | 77·78 | 41·67 | 14·58 | 63·89 | 14·58 | 20·83 | 69·0 | 38·9 |
|  | Werkgroep vterinair antibioticumbeleid - formularium geit [119] | 77·78 | 41·67 | 14·58 | 63·89 | 14·58 | 20·83 | 69·0 | 38·9 |
|  | Werkgroep vterinair antibioticumbeleid - formularium varken [120] | 77·78 | 41·67 | 14·58 | 63·89 | 14·58 | 20·83 | 69·0 | 38·9 |
|  | Richtlijn toepassen van antimicrobiële middelen [121] | 63·89 | 86·11 | 83·33 | 41·67 | 20·83 | 45·83 | 108·0 | 56·9 |
| New Zealand | Guide to prudent use of antimicrobial agents in pigs [122] | 75·00 | 38·89 | 16·67 | 83·33 | 10·42 | 4·17 | 69·5 | 38·1 |
|  | Antibiotic judicious use guidelines for the New Zealand veterinary profession in dairy [123] | 97·22 | 61·11 | 17·71 | 83·33 | 10·42 | 4·17 | 78·0 | 45·7 |
|  | Antibiotic judicious use guidelines for the New Zealand veterinary profession in equine [124] | 80·56 | 52·78 | 16·67 | 77·78 | 6·25 | 4·17 | 71·0 | 39·7 |
| Norway | Terapianbefaling: bruk av antibakterielle midler til produksjonsdyr [125] | 38·89 | 19·44 | 15·63 | 91·67 | 14·58 | 4·17 | 61·5·0 | 30·7 |
| Poland | Kodeks rozważnego stosowania produktów leczniczych przeciwdrobnoustrojowych przez lekarzy weterynarii [126] | 25·00 | 16·67 | 7·29 | 33·33 | 0·00 | 4·17 | 40·5·0 | 14·4 |
| Portugal | Utilização prudente de antimicrobianos em animais - vacas leiteiras [127] | 38·89 | 16·67 | 7·29 | 41·67 | 2·08 | 4·17 | 45·0 | 18·5 |
| South Africa*2 | Guidelines for the use of antimicrobials in the South African pig industry [128] | 83·33 | 5·56 | 6·25 | 44·44 | 12·50 | 4·17 | 53·5 | 26·0 |
|  | Technical guidelines for responsible and prudent use of antimicrobials in veterinary medicine in South Africa [129] | 52·78 | 25·00 | 7·29 | 36·11 | 10·42 | 4·17 | 50·0 | 22·6 |
| Spain | Recomendaciones para un uso prudente de los antibióticos en ganado bovino lechero [130] | 58·33 | 22·22 | 9·38 | 52·78 | 8·33 | 4·17 | 54·0 | 25·9 |
|  | Guía de uso responsable de medicamentos veterinarios: ovino y caprino [131] | 72·22 | 47·22 | 17·71 | 61·11 | 20·83 | 25·00 | 72·0 | 40·7 |
|  | Guía de uso responsable de medicamentos veterinarios: bovino [132] | 69·44 | 50·00 | 17·71 | 61·11 | 20·83 | 25·00 | 72·0 | 40·7 |
|  | Guía de uso responsable de medicamentos veterinarios: equinos [133] | 69·44 | 50·00 | 17·71 | 61·11 | 20·83 | 25·00 | 72·0 | 40·7 |
|  | Guía de uso responsable de medicamentos veterinarios: porcino [134] | 69·44 | 47·22 | 17·71 | 61·11 | 20·83 | 25·00 | 71·5 | 40·2 |
| Sweden | Sveriges Veterinärförbunds riktlinjer för antibiotikaanvändning till nötkreatur & gris [135] | 58·33 | 33·33 | 12·50 | 86·11 | 18·75 | 4·17 | 66·0 | 35·5 |
|  | Guidelines for the use of antibiotics in production animals - cattle, pigs, sheep and goats [136] | 72·22 | 47·22 | 15·63 | 77·78 | 14·58 | 4·17 | 70·0 | 38·6 |
|  | The Swedish Veterinary Association’s guidelines for the clinical use of antibiotics in the treatment of horses [137] | 80·56 | 27·78 | 14·58 | 77·78 | 18·75 | 4·17 | 68·5 | 37·3 |
| Switzerland | Guide thérapeutique pour les vétérinaires - utilisation prudente des antibiotiques: bovins, porcs, petits ruminants et camélidés du Nouveau Monde [138] | 91·67 | 50·00 | 17·71 | 77·78 | 22·92 | 4·17 | 77·0 | 44·0 |
|  | Directives concernant l'emploi judicieux des médicaments vétérinaires [139] | 50·00 | 50·00 | 12·50 | 36·11 | 2·08 | 4·17 | 54·5 | 25·8 |
| United Kingdom | BVA policy position on the responsible use of antimicrobials in food producing animals [140] | 50·00 | 22·22 | 16·67 | 66·67 | 10·42 | 4·17 | 59·0 | 28·4 |
| United States | The judicious use of medically important antimicrobial drugs in food-producing animals [141] | 50·00 | 25·00 | 17·71 | 33·33 | 4·17 | 4·17 | 52·5 | 22·4 |

The total score, in percentage, was calculated as the sum of the mean values obtained per item. The standardized quality of the guideline, represented as overall score, in percentage, was determined by the sum of all domain scores divided by 600 and multiplied by 100.

# **Appendix Table S7: Intraclass correlation coefficients, 95% confidence intervals, and interpretation for each AGREE II domain and item across the 42 appraised clinical guidelines.**

| AGREE II item per domain (Maximum score, %) | Median domain score, % (IQR) | ICC | CI 95% | | *p*-value | ICC interpretation |
| --- | --- | --- | --- | --- | --- | --- |
| Domain 1: Scope and purpose (97·22) | 69·44 (80·56 - 50·0) | 0·217 | -0·181 | 0·526 | 0·04 | Poor correlation |
| Item 1: The overall objective(s) of the guideline is (are) specifically described |  | 0·192 | -0·180 | 0·494 | 0·076 | Poor correlation |
| Item 2: The clinical question(s) covered by the guideline is (are) specifically described |  | 0·265 | -0·186 | 0·578 | 0·024 | Poor correlation |
| Item 3: The patients to whom the guideline is meant to apply are specifically described |  | 0·232 | -0·184 | 0·536 | 0·072 | Poor correlation |
| Domain 2: Stakeholder involvement (88·89) | 41·67 (50·00 - 25·00) | 0·587 | -0·058 | 0·818 | < 0·001 | Fair correlation |
| Item 4: The guideline development group includes individuals from all the relevant professional groups |  | 0·388 | -0·107 | 0·668 | 0·011 | Poor correlation |
| Item 5: The patients’ views and preferences have been sough |  | 0·780 | 0·757 | 0·884 | < 0·001 | Excellent correlation |
| Item 6: The target users of the guideline are clearly defined |  | 0·374 | -0·189 | 0·681 | 0·003 | Poor correlation |
| Domain 3: Rigor of development (83·33) | 15·63 (17·71 - 16·53) | 0·563 | 0·212 | 0·760 | 0·003 | Fair correlation |
| Item 7: Systematic methods were used to search for evidence |  | 0·905 | 0·826 | 0,949 | < 0·001 | Excellent correlation |
| Item 8: The criteria for selecting the evidence are clearly described |  | 0·755 | 0·546 | 0·867 | < 0·001 | Excellent correlation |
| Item 9: The strengths and limitations of the body of evidence are clearly described |  | 0·860 | 0·730 | 0·926 | < 0·001 | Excellent correlation |
| Item 10: The methods for formulating the recommendations are clearly described |  | 0·765 | 0·563 | 0·873 | < 0·001 | Excellent correlation |
| Item 11: The health benefits, side effects, and risks have been considered in formulating the recommendations |  | -0·669 | -1·950 | 0·086 | 0·983 | Poor correlation |
| Item 12: There is an explicit link between the recommendations and the supporting evidence |  | 0·172 | -0·196 | 0·472 | 0·133 | Poor correlation |
| Item 13: The guideline has been externally reviewed by experts prior to its publication |  | 0·273 | -0·277 | 0·595 | 0·137 | Poor correlation |
| Item 14: A procedure for updating the guideline is provided |  | 0·751 | 0·540 | 0·865 | < 0·001 | Excellent correlation |
| Domain 4: Clarity of presentation (91·67) | 63·89 (77·78 - 50·00) | 0·574 | 0·142 | 0·781 | < 0·001 | Fair correlation |
| Item 15: The recommendations are specific and unambiguous |  | 0·126 | -0·558 | 0·517 | 0·326 | Poor correlation |
| Item 16: The different options for management of the condition are clearly presented |  | 0·738 | 0·403 | 0·873 | < 0·001 | Good correlation |
| Item 17: Key recommendations are easily identifiable |  | 0·351 | -0·108 | 0·632 | 0·050 | Poor correlation |
| Domain 5: Applicability (22·92) | 12·50 (18·75 - 6·25) | -0·112 | -0568 | 0·276 | 0·715 | Poor correlation |
| Item 18: The guideline is supported with tools for application |  | 0·000 | -0·644 | 0·420 | 0·500 | Poor correlation |
| Item 19: The potential organizational barriers in applying the recommendations have been discussed |  | 0·000 | -0·102 | 0·146 | 0·500 | Poor correlation |
| Item 20: The potential cost implications of applying the recommendations have been considered |  | 0·000 | -0·616 | 0·413 | 0·500 | Poor correlation |
| Item 21: The guideline presents key review criteria for monitoring and/or audit purposes |  | -0·137 | -1·111 | 0·386 | 0·880 | Poor correlation |
| Domain 6: Editorial independence (50·00) | 4·17 (20·83 - 4·17) | 0·215 | -0·316 | 0·550 | 0·185 | Poor correlation |
| Item 22: The guideline is editorially independent from the funding body |  | 0·248 | -0·270 | 0·571 | 0·147 | Poor correlation |
| Item 23: Conflicts of interest of guideline development members have been recorded |  | 0·000 | -0·817 | 0·454 | 0·500 | Poor correlation |
| Total score (108·00) | 69·00 (72·00 - 54·50) | 0·504 | -0·150 | 0·774 | < 0·001 | Fair correlation |
| Overall score (64.20) | 38·00 (40·70 - 26·70) | 0.682 | 0.413 | 0.828 | < 0·001 | Good correlation |

CI: Confidence interval; ICC: Intraclass correlation coefficient; IQR: Interquartile range. For each domain and item, intraclass correlation coefficient (ICC), with a two-way mixed-effects model and 95% confidence interval (CI), was used to assess interrater reliability between appraisals. An ICC smaller than 0·40 was classified as a poor correlation, 0·40 to 0·59 as fair, 0·60 to 0·74 as good, and 0·75 to 1·0 as excellent.[142, 143] Analyses were performed with IBM SPSS version 29·0.

Note: Analysis was performed by calculating intraclass correlation coefficient using the two-way mixed-effects model and average measures.

# **Appendix Figure S1: AGREE II standardized scores of all guidelines (clinical and non-clinical) by domain.**

# **Appendix Table S8: List of international recommendations from the Codex Alimentarius (n=10) and from the Terrestrial Animal Health Code (n=10) that were assessed for compliance in the retrieved guidelines.**

| **Codex Alimentarius: CODE OF PRACTICE TO MINIMIZE AND CONTAIN FOODBORNE ANTIMICROBIAL RESISTANCE CXC 61-2005 FAO, 2021** |
| --- |
| - **Biosecurity, appropriate nutrition, vaccination, animal and plant/ crop best management practices,** and other alternative tools where appropriate, should be considered to reduce the need for use of antimicrobial agents. |
| - The decision to use antimicrobial agents should be based on **sound clinical judgement, experience, and treatment efficacy.** |
| - Medically important antimicrobials should be **prescribed**, administered, or applied only by, or under the direction of, **veterinarians**. |
| - Antimicrobial agents should be used as **legally authorized** and **following all applicable label directions;** except where specific legal exemptions apply. |
| - The choice of which antimicrobial agent to use should take into consideration relevant professional **guidelines, where available, results of antimicrobial susceptibility testing** of isolates from the production setting, where appropriate, and make adjustments to the antimicrobial agent selection based on clinical outcomes or when foodborne AMR risks become evident. |
| - Responsible and prudent use of antimicrobial agents does not include the use for **growth promotion** of antimicrobial agents that are considered medically important. |
| - Medically important antimicrobials should only be administered or applied for prevention/**prophylaxis** where professional oversight has identified well-defined and **exceptional** circumstances, appropriate dose and duration, based on clinical and epidemiological knowledge, consistent with the label, and in line with national legislation. |
| - When used for the control of disease/**metaphylaxis**, medically important antimicrobial agents should only be used on the basis of epidemiological and clinical knowledge and a diagnosis of a specific disease and follow **appropriate professional oversight**, dose, and duration. |
| - Countries could use additional risk management measures for medically important antimicrobials considered highest priority critically important as described in the ***WHO List of Critically Important Antimicrobials for Human Medicine*, the *OIE List of Antimicrobial Agents of Veterinary Importance***, or national lists, where available, including restrictions proportionate to risk and supported by scientific evidence. |
| - **Monitoring and surveillance of the use of antimicrobial agents and the incidence or prevalence,** and in particular trends, of foodborne AMR microorganisms and resistance determinants are among the critical factors to consider when developing risk management measures and evaluating the effectiveness of implemented risk management measure |
| **Terrestrial Animal Health Code (2023) Volume 1 CHAPTER 6.10. Responsible and prudent use of antimicrobial agents in veterinary medicine** |
| - Administer or prescribe antimicrobial agents only when necessary and taking into consideration the WOAH list of antimicrobial agents of veterinary importance; |
| - Make an appropriate choice of antimicrobial agents based on clinical experience and diagnostic laboratory information (pathogenic agent isolation, identification and antibiogram) where possible; |
| - Provide a detailed treatment protocol, including precautions and withdrawal times, especially when prescribing extra-label or off-label use. |
| - 2. Choosing antimicrobial agents |
| a) The expected efficacy of the treatment is based on: |
| i) the clinical experience of the veterinarians, their diagnostic insight and therapeutic judgement; |
| ii) diagnostic laboratory information (pathogenic agent isolation, identification and antibiogram); |
| iii) pharmacodynamics including the activity towards the pathogenic agents involved; |
| iv) the appropriate dosage regimen and route of administration; |
| v) pharmacokinetics and tissue distribution to ensure that the selected therapeutic agent is effective at the site of infection; |
| vi) the epidemiological history of the rearing unit, particularly in relation to the antimicrobial resistance profiles of the pathogenic agents involved. |
| - Should a first-line antimicrobial treatment fail or should the disease recur, a second line treatment should be based on the results of diagnostic tests. In the absence of such results, an appropriate antimicrobial agent belonging to a different class or sub-class should be used. |
| - In emergencies, a veterinarian may treat animals without recourse to an accurate diagnosis and antimicrobial susceptibility testing, to prevent the development of clinical disease and for reasons of animal welfare. |
| - Use of combinations of antimicrobial agents should be scientifically supported. Combinations of antimicrobial agents may be used for their synergistic effect to increase therapeutic efficacy or to broaden the spectrum of activity. |
| - A prescription for VMP containing antimicrobial agents should indicate precisely the dosage regimen, the withdrawal period (where applicable) and the amount of VMP containing antimicrobial agents to be provided, depending on the dosage and the number of animals to be treated.) |
| - The extra-label or off-label use of VMP containing antimicrobial agents may be permitted in appropriate circumstances and should be in agreement with the national legislation in force including the withdrawal periods to be used, as applicable. It is the veterinarian's responsibility to define the conditions of responsible use in such a case including the dosage regimen, the route of administration and the withdrawal period. |
| - Records on VMP containing antimicrobial agents should be kept in conformity with the national legislation. |

# **Appendix text S1: References.**

1. Cutler R, Gleeson B, Page SW, Norris JM, Browning GF. Antimicrobial prescribing guidelines for pigs. Aust Vet J. 2020;98(4):105-34. doi: 10.1111/avj.12940.

2. Batey R, Nilon P, Page SW, Browning GF, Norris JM. Antimicrobial prescribing guidelines for sheep. Aust Vet J. 2024;102(4):103-42. doi: 10.1111/avj.13310.

3. House JK, Izzo MM, Page SW, Browning GF, Norris JM. Antimicrobial prescribing guidelines for dairy cattle. Aust Vet J. 2024;102(4):143-86. doi: 10.1111/avj.13311.

4. Cusack P, Batterham T, Page SW, Browning GF, M. NJ. Antimicrobial prescribing guidelines for feedlot cattle. 2024.

5. Leitlinien für den sorgfältigen Umgang mit antibakteriell wirksamen Tierarzneimitteln [Guidelines for the careful handling of antibacterial veterinary medicinal products]. https://www.ooe-tgd.at/Mediendateien/AVN_20240228_AVN_2024_2_2.pdf (2024). Accessed February 12, 2024.

6. Mesures pour un bon usage des antibiotiques lors d’un traitement de groupe chez les bovins [Measures for the correct use of antibiotics during group treatment of bovines]. https://amcra.be/swfiles/files/Advies-groepsbehandeling-bij-rundvee-_Goedgekeurd-RvB-22112021_FR.pdf (2021). Accessed February 5, 2024.

7. Mesures pour un bon usage des antibiotiques lors d’un traitement de groupe des porcs [Measures for the correct use of antibiotics during group treatment of pigs]. https://www.amcra.be/swfiles/files/Finale-versie_na-goedkeuring-RvB_29012021_FR.pdf (2021). Accessed February 5, 2024.

8. Surveillance et utilisation des antibiotiques, y compris de ceux d’importance critique, chez les chevaux [Monitoring and use of antibiotics, including those of critical importance, in horses. Current situation and recommendations for the future.]. https://www.amcra.be/swfiles/files/Avis%20chevaux%20utilisation%20CIA_FR_Approuv%C3%A9%20CA%20AMCRA%2027.02.2020_403.pdf (2020). Accessed February 5, 2024.

9. Lentz CAM: Atualização sobre Uso Racional de Antimicrobianos e Boas Práticas de Produção [Update on the Rational Use of Antimicrobials and Good Production Practices]. https://www.gov.br/agricultura/pt-br/assuntos/insumos-agropecuarios/insumos-pecuarios/resistencia-aos-antimicrobianos/publicacoes/Apostila_AtualizaosobreUsoRacionaldeAntimicrobianoseBoasPrticasdeProduo.pdf (2022). Accessed February 12, 2024.

10. Ръководство за разумна и отговорна употреба на антимикробни средства във ветеринарната медицина и практика [Guidelines for the reasonable and responsible use of antimicrobial agents in veterinary medicine and practice]. https://www.mzh.government.bg/media/filer_public/2023/04/26/_za_razumna_i_otgovorna_upotreba_na_antimikrobni_sredstva_final.pdf (2023). Accessed February 12, 2024.

11. CVMA Antimicrobial Prudent Use Guidelines 2008 for Beef Cattle, Dairy Cattle, Poultry and Swine. https://worldvet.org/uploads/docs/cvma_antimicrobial_prudent_use_guidelines_2008_for_beef_dairy_poultry_swine.pdf (2008). Accessed February 1, 2024.

12. Rodríguez M, Cárcamo AP, Fonseca AM, Grizales AMP, Sierra EB, Sabogal AIR, et al.: Manual de Buenas Prácticas en el uso de Medicamentos Veterinarios en la Porcicultura [Manual for Good Practices for the use of Veterinary Medicines in Pig Farming]. https://www.ica.gov.co/getattachment/Areas/Pecuaria/Servicios/Inocuidad-en-las-Cadenas-Agroalimentarias/LISTADO-DE-PREDIOS-CERTIFICADOS-EN-BPG/Manual-Uso-MEDICAMENTOS-1.pdf.aspx?lang=es-CO (2019). Accessed February 1, 2024.

13. Castañeda DCF, B. MJF, Godoy MPD: Guía de uso prudente de antibióticos en la producción de leche a partir del modelo de salud de hato [Guide to the prudent use of antibiotics in milk production from the herd health model]. https://editorial.agrosavia.co/index.php/publicaciones/catalog/view/101/87/856-1 (2020). Accessed February 1, 2024.

14. Guía de uso responsable de medicamentos veterinarios en bovinos [Guide for the responsible use of veterinary medicines in bovines]. https://www.senasa.go.cr/informacion/centro-de-informacion/informacion/manuales-de-buenas-practicas/3659-guia-de-uso-responsable-de-medicamentos-veterinarios-en-bovinos (2018). Accessed February 1, 2024.

15. Guideline for Prescribing Antimicrobial for Pigs. https://foedevarestyrelsen.dk/Media/638225168488658373/Antimicrobial%20guideline%20for%20pigs%20Denmark%202018.pdf (2018). Accessed February 1, 2024.

16. Kalmus P, Aasmäe B: Juhend Antibiootikumide Kasutamiseks Põllumajandusloomadel (Clinical guidelines, clinical practice guidelines) [Guidelines for the use of Antibiotics in Farm Animals (Clinical guidelines, clinical practice guidelines)]. https://pta.agri.ee/media/2231/download (2020). Accessed February 12, 2024.

17. Recommendations for the use of antimicrobials in the treatment of the most significant infectious and contagious diseases in animals. https://www.ruokavirasto.fi/globalassets/viljelijat/elaintenpito/elainten-laakitseminen/hallittu_laakekekaytto/mikrobilaakekaytonperiaatteet/mikrobilaakkeiden_kayttosuositukset_en.pdf (2018). Accessed February 1, 2024.

18. Guide de bonnes pratiques de l’antibiothérapie vétérinaire à l’usage des vétérinaires [Guide to good practice in veterinary antibiotic therapy for veterinarians]. https://www.veterinaire.fr/system/files/files/2021-12/Guide_de_bonnes_pratiques_de_l_antibiotherapie_veterinaire_a_l_usage_des_veterinaires.pdf (2009). Accessed July 22, 2024.

19. Guidelines for the prudent use of veterinary antimicrobial drugs - with notes for guidance. https://www.google.com/url?sa=t&rct=j&q=&esrc=s&source=web&cd=&ved=2ahUKEwjB34-N67yEAxW7gP0HHUuQAxwQFnoECA8QAQ&url=https%3A%2F%2Fwww.bundestieraerztekammer.de%2Fbtk%2Fdownloads%2Fantibiotika%2FAB_Leitlinien2015_EN.pdf&usg=AOvVaw2Yujei7csjrlJre1G5pFQ9&opi=89978449https://www.bundestieraerztekammer.de/btk/downloads/antibiotika/AB_Leitlinien2015_EN.pdf (2015). Accessed February 1, 2024.

20. Εγχειριδιο εμποριασ, προμηθειασ και χρησησ κτηνιατρικων φαρμακευτικων προϊοντων [Handbook on marketing, supply and use of veterinary medicinal products]. https://www.minagric.gr/images/stories/docs/agrotis/ktiniatrika_Farmaka/enxeiridio_ktin_farmakon310521.pdf (2020). Accessed February 12, 2024.

21. Az antibiotikum- kezelés minimumkövetelményeire vonatkozó útmutató [Guidelines for minimum requirements for antibiotic treatment]. https://portal.nebih.gov.hu/documents/10182/21360/Antibiotikumfelhasznalas-csokkentesi_terv.pdf (2022). Accessed February 12, 2024.

22. Asean guidelines for the prudent use of antimicrobials in livestock. https://asean.org/wp-content/uploads/2021/09/ASEAN-Guidelines-for-Prudent-Use-of-Antimicrobials-in-Livestock.pdf (2017). Accessed February 12, 2024.

23. Nasrullah, Rasa FST, Isriyanthi NMR, Ratnasari YE, Fauzi M, Utomo GB, et al.: Pedoman Umum Penggunaan Antibiotik di Bidang Peternakan dan Kesehatan Hewan [General Guidelines - Use of Antibiotics in the Field of Animal Husbandry and Animal Health]. https://repository.pertanian.go.id/server/api/core/bitstreams/6f3d4864-9d19-4d06-9e20-7302b7c2df1a/content (2021). Accessed February 12, 2024.

24. Code of Good Practice Regarding the Responsible Prescribing and Use of Antibiotics in Farm Animals. https://www.apha.ie/Documents/Code%20of%20Good%20Practice%20Regarding%20the%20Responsible%20Prescribing%20and%20Use%20of%20Antibiotics%20in%20Farm%20Animals.pdf (2018). Accessed February 5, 2024.

25. Code of Good Practice Regarding the Responsible Use of Antimicrobials on Pig Farms. https://animalhealthireland.ie/assets/uploads/2021/06/Code-of-Good-Practice-for-Responsible-Use-of-AMs-on-Pig-Farms.pdf?dl=1https://www.google.com/url?sa=t&source=web&rct=j&opi=89978449&url= (2019). Accessed February 5, 2024.

26. Code of Good Practice Regarding the Responsible Use of Antimicrobials on Dairy Farms. https://animalhealthireland.ie/assets/uploads/2021/06/Code-of-Good-Practice-for-Responsible-Use-of-AMs-on-Dairy-Farms.pdf?dl=1 (2019). Accessed February 5, 2024.

27. Code of Good Practice regarding responsible use of Antimicrobials on Suckler and Beef Farms. https://www.apha.ie/Documents/Guidelines_Beef_Sept_2020.pdf (2020). Accessed February 5, 2024.

28. Code of Good Practice Regarding the Responsible Use of Antimicrobials on Sheep Farms. https://www.apha.ie/Documents/Code%20of%20Good%20Practice%20Regarding%20the%20Responsible%20Prescribing%20and%20Use%20of%20Antibiotics%20in%20Farm%20Animals.pdf (2020). Accessed February 5, 2024.

29. Equine Antimicrobial Use Guidelines. https://assets.gov.ie/246259/efd59850-1944-452f-8725-958f67fe384d.pdf (2022). Accessed February 5, 2024.

30. Vademecum per un uso prudente e razionale degli antibiotici nelle produzioni zootecniche [Vademecum for the prudent and rational use of antibiotics in livestock production]. https://www.izslt.it/crab/wp-content/uploads/sites/8/2018/07/Vademecum-uso-razionale-e-prudente-degli-antibiotici.pdf (2018). Accessed February 12, 2024.

31. Linee guida per l’uso prudente degli antimicrobici negli allevamenti zootecnici per la prevenzione dell’antimicrobico-resistenza e proposte alternative [Guidelines for the prudent use of antimicrobials in zootechnical farming for the prevention of antimicrobial resistance and alternative proposals]. https://www.salute.gov.it/imgs/C_17_pubblicazioni_2782_allegato.pdf (2018). Accessed February 12, 2024.

32. Bassi P, Casadio C, Luppi A, Giuseppe M, Merialdi G, Padovani A, et al.: Linee Guida - Uso prudente degli antibiotici nell’allevamento suino [Guidelines - Prudent use of antibiotics in pig farming]. https://www.alimenti-salute.it/sites/default/files/LG_AMR_SUINO_rev._2022.pdf (2022). Accessed February 12, 2024.

33. Arrigoni N, Bassi P, Maragno D, Padovani A, Trambajolo G: Linee guida - Uso prudente dell'antibiotico nell'allevamento bovino da latte [Guidelines Prudent use of antibiotics in dairy cattle breeding]. https://www.salute.gov.it/imgs/C_17_pubblicazioni_3347_allegato.pdf (2023). Accessed February 12, 2024.

34. 動物用抗菌剤の 『責任ある慎重使用』を 進めるために [To promote the responsible and prudent use of veterinary antimicrobial agents]. https://www.maff.go.jp/j/syouan/tikusui/yakuzi/pdf/vet_panf_prudent_use.pdf (2000). Accessed February 12, 2024.

35. 畜産物生産における動物用抗菌性物質製剤の 慎重使用に関する基本的な考え方 [Basic concept regarding the prudent use of animal antibacterial preparations in livestock product production]. https://www.maff.go.jp/j/syouan/tikusui/yakuzi/pdf/prudent_use.pdf (2013). Accessed February 12, 2024.

36. 소 항생제 처방 가이드라인 [Bovine antibiotic prescribing guidelines]. https://ebook.qia.go.kr/20210104_100444/ (2020). Accessed February 12, 2024.

37. 돼지 항생제 처방 가이드라인 [Swine antibiotic prescribing guidelines]. https://ebook.qia.go.kr/20210104_100841/ (2020). Accessed February 12, 2024.

38. 산업동물 수의사를 위한 항생제 길잡이 [Antibiotic Guide for Veterinarians of Industrial Animals]. http://qia.go.kr/downloadwebQiaCom.do?id=44774 (2022). Accessed February 12, 2024.

39. Mičules G: Vadlīnijas antimikrobiālās rezistences attīstības ierobežošanai lauksaimnieciskās ražošanas posmā un veterinārmedicīnas praksē Latvijā [Guidelines for limiting the development of antimicrobial resistance during agricultural production in veterinary practice in Latvia]. https://lvb.lv/wp-content/uploads/2021/12/Vadlinijas.VPP_.pdf (2018). Accessed February 12, 2024.

40. Empfehlungen für den sachgemäßen Einsatz von Antibiotika in der Tiergesundheit [Recommendations for the appropriate use of antibiotics in animal health]. https://agriculture.public.lu/dam-assets/veroeffentlichungen/broschueren/tiere/2022-einsatz-antibiotika-tiergesundheit.pdf (2022). Accessed February 5, 2024.

41. Guía para el Buen Uso de Productos Farmacéuticos Veterinarios [Guide for the Good Use of Veterinary Pharmaceutical Products]. https://acsaa.com.mx/wp-content/uploads/2022/02/Gu_a_para_buen_uso_de_productos_farmaceuticoss.pdf (2021). Accessed February 1, 2024.

42. Guía Del Buen Uso De Antimicrobianos En Cerdos [Guide to the Good Use of Antimicrobials in Pigs]. https://www.gob.mx/cms/uploads/attachment/file/837407/GU_A_DEL_BUEN_USO_DE_ANTIMICROBIANOS_EN_CERDOS.pdf (2023). Accessed February 1, 2024.

43. Richtlijn Toepassen van antimicrobiële middelen [Guideline for the use of antimicrobials]. https://www.knmvd.nl/app/uploads/2022/03/150513-Richtlijn-TAM-definitief-versie-1.1.pdf (2015). Accessed February 5, 2024.

44. van Duijkeren E, Hartog PA, van Hout AJ, Kanters MJF, van Nes A, Schyns MAR: Werkgroep Vterinair Antibioticumbeleid - Formularium Varken [Working Group on Veterinary Antibiotic Policy - Pig Formulary]. https://www.knmvd.nl/app/uploads/sites/4/2019/09/formularium-varken_230919.pdf (2019). Accessed February 5, 2024.

45. van den Bosch MBW, Bouwman S, van den Brom R, Dijkstra E, van der Hidjen M, van den Oord P, et al.: Werkgroep Vterinair Antibioticumbeleid - Formularium Geit [Veterinary Antibiotic Policy Working Group - Goat Formulary]. https://www.knmvd.nl/app/uploads/sites/4/2020/09/formularium-geit-versie-1.1_220920.pdf (2019). Accessed February 5, 2024.

46. D'Hoe K, Last WJ, Mölder P, Vendrig JC: Formularium vleeskalveren en vleesvee [Formulary for veal calves and beef cattle]. https://www.knmvd.nl/app/uploads/sites/4/2018/09/170315-wvab-formularium-vleeskalveren-en-vleesvee_definitief.docx.pdf (2019). Accessed February 5, 2024.

47. van den Bosch MBW, Bouwman S, van den Brom R, Dijkstra E, van der Hidjen M, van den Oord P, et al.: Formularium Kleine Herkauwers - Schaap [Formulary of small ruminants - sheep]. https://www.knmvd.nl/app/uploads/sites/4/2019/07/190619-formularium-schaap-definitief.pdf (2019). Accessed February 5, 2024.

48. Bakker J, van Duijkeren E, Ensink JM, Panhuijzen JJA, van Oldruitenborgh-Oosterbaan MMS, Stout TAE, et al.: Werkgroep Vterinair Antibioticumbeleid - Formularium Paard [Working Group on Veterinary Antibiotic Policy - Horse Formulary]. https://www.knmvd.nl/app/uploads/sites/4/2021/03/wvab-formularium-paard-2016-versie-1.3.pdf (2021). Accessed February 12, 2024.

49. Bierens JJM, van Beijnum LM, Dierikx CM, Palevliet JM, van Rossum nRJW, Vending JC: Werkgroep Vterinair Antibioticumbeleid - Formularium Melkvee [Working Group on Veterinary Antibiotic Policy - Dairy Cattle Formulary. https://www.knmvd.nl/app/uploads/sites/4/2023/06/230531-formularium-melkvee-versie-1.931.pdf (2023). Accessed June 16, 2024.

50. Antibiotic judicious use guidelines for the New Zealand veterinary profession in Dairy. https://www.amrvetcollective.com/assets/guidelines/guide_dairy.pdf (2018). Accessed February 1, 2024.

51. Guide to prudent use of antimicrobial agents in Red meat production. https://www.amrvetcollective.com/assets/guidelines/guide_redmeat.pdf (2018). Accessed February 1, 2024.

52. Antibiotic judicious use guidelines for the New Zealand veterinary profession in Equine. https://www.amrvetcollective.com/assets/guidelines/guide_equine.pdf (2018). Accessed February 1, 2024.

53. Guide to prudent use of antimicrobial agents in Pigs. https://www.amrvetcollective.com/assets/guidelines/AMR_Guide_Pigs.pdf (2020). Accessed February 1, 2024.

54. Terapianbefaling: Bruk av antibakterielle midler til produksjonsdyr [Therapeutic recommendation: Use of antibacterial agents for production animals]. https://www.dmp.no/globalassets/documents/veterinarmedisin/terapianbefalinger/terapianbefaling---bruk-av-antibakterielle-midler-til-produksjonsdyr.pdf (2022). Accessed February 1, 2024.

55. Wysocki M: Kodeks Rozważnego Stosowania Produktów Leczniczych Przeciwdrobnoustrojowych Przez Lekarzy Weterynarii [Code of the prudent use of antimicrobial medicinal products by veterinarians]. https://www.izbawetbial.pl/files/B202001.pdf (2020). Accessed February 12, 2024.

56. Manual de Boas Práticas: Utilização de Antimicrobianos em Animais Produtores de Géneros Alimentícios [Manual of Good Practices for the Use of Antimicrobials in Food-Producing Animals]. https://saaf.dgadr.gov.pt/images/DGAV_ManualBoasPraticas_Antimicrobianos.pdf (2021). Accessed February 12, 2024.

57. Ramalho FA: Utilização prudente de antimicrobianos em animais - Vacas leiteiras [Prudent use of antimicrobials in animals dairy cows]. https://www.dgav.pt/wp-content/uploads/2023/07/HIPRA-UNIVERSITY_Filomena-Ramalho_MEDICAMENTOS-VETERINARIOS_.pdf (2023). Accessed February 1, 2024.

58. Ghidul naţional privind utilizarea prudentă a antimicrobienelor în medicina veterinară [National guidelines on the prudent use of antimicrobial in veterinary medicine]. http://www.ansvsa.ro/download/antimicrobieni/Ghidul-national-privind-utilizarea-prudenta-a-antimicrobienelor-in-medicina-veterinara-actualizat.pdf (2020). Accessed February 12, 2024.

59. Splošna priporočila za uporabo antibiotikov v veterinarske namene [General recommendations for the use of antibiotics for veterinary purposes]. https://www.gov.si/assets/organi-v-sestavi/UVHVVR/Zdravila/Zdravila-v-veterinarski-medicini/splosna-veterinarski-namen.pdf Accessed February 12, 2024.

60. Technical Guidelines for Responsible and Prudent Use of Antimicrobials in Veterinary Medicine in South Africa. https://worldvet.org/uploads/docs/technical_guidelines_for_the_prudent_use_of_antimicrobials.pdf (2002). Accessed February 1, 2024.

61. Guidelines for the use of antimicrobials in the South African pig industry. https://www.sava.co.za/wp-content/uploads/2017/05/Antibiotic-Guidelines-Pig-Industry-Ver-20161027-1.pdf (2016). Accessed February 1, 2024.

62. Guía de Uso Responsable de Medicamentos Veterinarios: Bovino [Guide for the Responsible Use of Veterinary Medicines: Bovine]. https://www.vetresponsable.es/vetresponsable/guias-de-uso-responsable-por-especie-animal/bovino_3930_340_4076_0_1_in.html (2017). Accessed February 1, 2024.

63. Guía de Uso Responsable de Medicamentos Veterinarios: Equinos [Guide for the Responsible Use of Veterinary Medicines: Equines]. https://www.vetresponsable.es/vetresponsable/guias-de-uso-responsable-por-especie-animal/equino_3929_340_4075_0_1_in.html (2017). Accessed February 1, 2024.

64. Guía de Uso Responsable de Medicamentos Veterinarios: Porcino [Guide for the Responsible Use of Veterinary Medicines: Porcine]. https://www.vetresponsable.es/vetresponsable/guias-de-uso-responsable-por-especie-animal/porcino_3928_340_4074_0_1_in.html (2017). Accessed February 1, 2024.

65. Recomendaciones para un uso prudente de los antibióticos en ganado bovino lechero [Recommendations for prudent use of antibiotics in dairy cattle]. https://neiker.eus/newsletters/documentos/guia-veterinario-es.pdf (2021). Accessed February 5, 2024.

66. Guía de Uso Responsable de Medicamentos Veterinarios: Ovino y caprino [Guide for the Responsible Use of Veterinary Medicines: Sheep and goats]. https://www.vetresponsable.es/vetresponsable/guias-de-uso-responsable-por-especie-animal/ovino-y-caprino_3931_340_4077_0_1_in.html (2021). Accessed February 1, 2024.

67. The Swedish Veterinary Association’s Guidelines for the clinical use of antibiotics in the treatment of horses. https://www.svf.se/media/tztkij4b/guidelines-antibiotics-in-horses.pdf (2013). Accessed February 1, 2024.

68. Guidelines for the use of antibiotics in production animals - Cattle, pigs, sheep and goats. https://www.svf.se/media/vd5ney4l/svfs-riktlinje-antibiotika-till-produktionsdjur-eng-2017.pdf (2017). Accessed February 1, 2024.

69. Sveriges Veterinärförbunds Riktlinjer För Antibiotikaanvändning Till Nötkreatur & Gris [Swedish Veterinary Association Guidelines for Antibiotic Use in Cattle and Pigs]. https://www.svf.se/media/segp21ok/abriktlinjer-no-tkreatur-och-gris-rev2019.pdf#/media/media/edit/11574 (2019). Accessed February 1, 2024.

70. Guide thérapeutique pour les vétérinaires - Utilisation prudente des antibiotiques: Bovins, Porcs, Petits Ruminants et Camélidés du Nouveau Monde [Therapeutic guide for veterinarians - Prudent use of antibiotics: cattle, pigs, small ruminants and New World camelids]. https://www.blv.admin.ch/dam/blv/fr/dokumente/tiere/tierkrankheiten-und-arzneimittel/tierarzneimittel/therapieleitfaden.pdf.download.pdf/therapieleitfaden-fr.pdf (2022). Accessed February 1, 2024.

71. Directives concernant l'emploi judicieux des médicaments vétérinaires [Guidelines for the judicious use of veterinary drugs]. https://www.gstsvs.ch/fileadmin/user_upload/GST-SVS/Publikationen/Richtlinien_Umgang_TAM_f.pdf (2022). Accessed February 1, 2024.

72. Code Of Practice For Control Of The Use Of Veterinary Drugs. https://faolex.fao.org/docs/pdf/tha176163.pdf (2009). Accessed February 12, 2024.

73. Code of Practice on the responsible use of animal medicines on the farm. https://www.gov.uk/government/publications/responsible-use-of-animal-medicines-on-the-farm/code-of-practice-on-the-responsible-use-of-animal-medicines-on-the-farm (2014). Accessed February 1, 2024.

74. Practical Guide to Responsible Use of Antibiotics on Pig Farms. https://ruma.org.uk/wp-content/uploads/2022/08/Responsible-Use-Of-Antimicrobials-in-Pig-Production.pdf (2018). Accessed February 1, 2024.

75. BVA policy position on the responsible use of antimicrobials in food producing animals. https://www.bva.co.uk/media/1161/bva-policy-position-on-the-responsible-use-of-antimicrobials-in-food-producing-animals-1.pdf (2019). Accessed February 1, 2024.

76. Locatt F, Duncan J, Hinde D, King L: Industry guidance document for veterinary surgeons and farmers on responsible use of antibiotics in sheep. https://ruma.org.uk/wp-content/uploads/2022/08/Responsible-Use-of-Antimicrobials-in-Sheep-Production.pdf (2019). Accessed February 1, 2024.

77. Using medicines responsibly: As little as possible, but as much as necessary. https://projectblue.blob.core.windows.net/media/Default/Beef%20&%20Lamb/Using%20Medicines3784_200818_WEB.pdf (2020). Accessed February 1, 2024.

78. Guidelines on Responsible Use of Antimicrobials in Dry Cow Strategies. https://ruma.org.uk/wp-content/uploads/2022/08/Guidelines-on-Responsible-Use-of-Antimicrobials-in-Dry-Cow-Strategies.pdf (2022). Accessed February 1, 2024.

79. Guidelines for Responsible use of antimicrobials in cattle production. https://www.ruma.org.uk/wp-content/uploads/2023/06/RUMA_antimicrobial_long_cattle_-2022-revisions-Final-.pdf (2022). Accessed February 1, 2024.

80. The Judicious Use of Medically Important Antimicrobial Drugs in Food-Producing Animals. https://www.fda.gov/media/79140/download (2012). Accessed February 1, 2024.

81. Guidelines for Veterinarians: Judicious Use of Antimicrobials in Livestock; Guidance for Industry. https://www.cdfa.ca.gov/ahfss/aus/docs/Guidelines_Veterinarians_Judicious_Use_of_Antimicrobials_Livestock.pdf (2020). Accessed February 1, 2024.

82. Importance Ratings and Summary of Antibacterial Uses in Human and Animal Health in Australia. https://www.amr.gov.au/sites/default/files/2022-10/importance-ratings-and-summary-of-antibacterial-uses-in-human-and-animal-health-in-australia.pdf (2018). Accessed on March 5, 2024.

83. Critically Important Antimicrobials for Human Medicine. https://iris.who.int/bitstream/handle/10665/312266/9789241515528-eng.pdf?sequence=1 (2019). Accessed on March 5, 2024.

84. OIE List of Antimicrobial Agents of Veterinary Importance. https://www.woah.org/app/uploads/2021/06/a-oie-list-antimicrobials-june2021.pdf (2021). Accessed on March 5, 2024.

85. Categorisation of Antibiotics in the European Union. https://www.ema.europa.eu/en/documents/report/categorisation-antibiotics-european-union-answer-request-european-commission-updating-scientific-advice-impact-public-health-and-animal-health-use-antibiotics-animals_en.pdf (2019). Accessed August 23, 2024.

86. Categorization of Antimicrobial Drugs Based on Importance in Human Medicine. https://www.canada.ca/en/health-canada/services/drugs-health-products/veterinary-drugs/antimicrobial-resistance/categorization-antimicrobial-drugs-based-importance-human-medicine.html (2009). Accessed on March 5, 2024.

87. OIE Lista de agentes antimicrobianos de importancia para la medicina veterinaria. https://www.woah.org/fileadmin/Home/esp/Our_scientific_expertise/docs/pdf/AMR/E_OIE_Lista_antimicrobianos_Julio2019.pdf (2019). Accessed on March 5, 2024.

88. Retningslinjer for brug af antibiotika til kvæg i Danmark [Guidelines for the use of antibiotics for cattle in Denmark]. https://www.ddd.dk/media/2331/retningslinjer-for-brug-af-antibiotika-kvaeg.pdf (2013). Accessed February 1, 2024.

89. Critically Important Antimicrobials for Human Medicine. https://iris.who.int/bitstream/handle/10665/251715/9789241511469-eng.pdf?sequence=1 (2016). Accessed on March 5, 2024.

90. OIE List of Antimicrobial Agents of Veterinary Importance. https://www.woah.org/fileadmin/Home/eng/Our_scientific_expertise/docs/pdf/Eng_OIE_List_antimicrobials_May2015.pdf (2015). Accessed on March 5, 2024.

91. Critically Important Antimicrobials for Human Medicine. https://iris.who.int/bitstream/handle/10665/255027/9789241512220-eng.pdf?sequence=1 (2017). Accessed on March 5, 2024.

92. European Medicines Agency (EMA). Categorisation of antibiotics for use in animals: For prudent and responsible use. https://www.ema.europa.eu/en/documents/report/infographic-categorisation-antibiotics-use-animals-prudent-and-responsible-use_en.pdf Accessed March 4, 2024.

93. OIE List of Antimicrobial Agents of Veterinary Importance. https://www.woah.org/fileadmin/Home/eng/Our_scientific_expertise/docs/pdf/Eng_OIE_List_antimicrobials_May2015.pdf (2015). Accessed August 20, 2024.

94. OIE List of Antimicrobial Agents of Veterinary Importance. https://www.woah.org/app/uploads/2021/03/a-oie-list-antimicrobials-may2018.pdf (2018). Accessed on March 5, 2024.

95. WVAB – richtlijn classificatie van veterinaire antimicrobiële middelen [WVAB – Guidelines for the classification of antimicrobials for veterinary]. https://www.knmvd.nl/app/uploads/sites/4/2021/08/WVAB-richtlijn-3.4-definitief_160821.pdf (2021). Accessed on March 5, 2024.

96. Critically Important Antimicrobials for Human Medicine. https://iris.who.int/bitstream/handle/10665/77444/9789241501439_eng.pdf?sequence=1 (2011). Accessed on March 5, 2024.

97. Critically Important Antimicrobials for Human Medicine. https://iris.who.int/bitstream/handle/10665/77376/9789241504485_eng.pdf?sequence=1 (2012). Accessed on March 5, 2024.

98. Categorisation of antibiotics in the European Union. https://www.ema.europa.eu/en/documents/report/categorisation-antibiotics-european-union-answer-request-european-commission-updating-scientific-advice-impact-public-health-and-animal-health-use-antibiotics-animals_en.pdf (2020). Accessed on March 5, 2024.

99. Critically Important Antimicrobials for Human Medicine for risk management stratagies of non-human use. https://iris.who.int/bitstream/handle/10665/43330/9241593601_eng.pdf?sequence=1 (2005). Accessed on March 5, 2024.

100. Critically Important Antimicrobials for Human Medicine: Categorization for the Development of Risk Management Strategies to contain Antimicrobial Resistance due to Non-Human Antimicrobial Use. https://iris.who.int/bitstream/handle/10665/43765/9789241595742_eng.pdf?sequence=1 (2007). Accessed on March 5, 2024.

101. WHO List of Medically Important Antimicrobials. https://cdn.who.int/media/docs/default-source/gcp/who-mia-list-2024-lv.pdf?sfvrsn=3320dd3d_2 (2024). Accessed on March 5, 2024.

102. Cusack P, Batterham T, Page SW, Browning GF, M. NJ: Antimicrobial prescribing guidelines for feedlot cattle. https://animalmedicinesaustralia.org.au/wp-content/uploads/2024/05/antimicrobial-prescribing-guidelines-feedlot-cattle-21-03-24.pdf (2024). Accessed on February 12, 2024.

103. Leitlinien für den sorgfältigen umgang mit antibakteriell wirksamen tierarzneimitteln [Guidelines for the careful handling of antibacterial veterinary medicinal products]. https://www.ooe-tgd.at/Mediendateien/AVN_20240228_AVN_2024_2_2.pdf (2024). Accessed on February 12, 2024.

104. Surveillance et utilisation des antibiotiques, y compris de ceux d’importance critique, chez les chevaux [Monitoring and use of antibiotics, including those of critical importance, in horses]. https://www.amcra.be/swfiles/files/Avis%20chevaux%20utilisation%20CIA_FR_Approuv%C3%A9%20CA%20AMCRA%2027.02.2020_403.pdf (2020). Accessed on February 5, 2024.

105. Antimicrobial prudent use guidelines for beef cattle, dairy cattle, poultry, and swine. https://worldvet.org/uploads/docs/cvma_antimicrobial_prudent_use_guidelines_2008_for_beef_dairy_poultry_swine.pdf (2008). Accessed on February 1, 2024.

106. Guideline for prescribing antimicrobial for pigs. https://foedevarestyrelsen.dk/Media/638225168488658373/Antimicrobial%20guideline%20for%20pigs%20Denmark%202018.pdf (2018). Accessed on February 1, 2024.

107. Kalmus P, Aasmäe B: Juhend antibiootikumide kasutamiseks põllumajandusloomadel [Guidelines for the use of antibiotics in farm animals]. https://pta.agri.ee/media/2231/download (2020). Accessed on February 12, 2024.

108. Recommendations for the use of antimicrobials in the treatment of the most significant infectious and contagious diseases in animals. https://www.ruokavirasto.fi/globalassets/viljelijat/elaintenpito/elainten-laakitseminen/hallittu_laakekekaytto/mikrobilaakekaytonperiaatteet/mikrobilaakkeiden_kayttosuositukset_en.pdf (2018). Accessed on February 1, 2024.

109. Guidelines for the prudent use of veterinary antimicrobial drugs -with notes for guidance-. https://www.google.com/url?sa=t&rct=j&q=&esrc=s&source=web&cd=&ved=2ahUKEwjB34-N67yEAxW7gP0HHUuQAxwQFnoECA8QAQ&url=https%3A%2F%2Fwww.bundestieraerztekammer.de%2Fbtk%2Fdownloads%2Fantibiotika%2FAB_Leitlinien2015_EN.pdf&usg=AOvVaw2Yujei7csjrlJre1G5pFQ9&opi=89978449https://www.bundestieraerztekammer.de/btk/downloads/antibiotika/AB_Leitlinien2015_EN.pdf (2015). Accessed on February 1, 2024.

110. Az antibiotikum - kezelés minimumkövetelményeire vonatkozó útmutató [Guidelines for minimum requirements for antibiotic treatment]. https://portal.nebih.gov.hu/documents/10182/21360/Antibiotikumfelhasznalas-csokkentesi_terv.pdf (2022). Accessed on February 12, 2024.

111. 산업동물 수의사를 위한 항생제 길잡이 [Antibiotic guide for veterinarians of industrial animals]. http://qia.go.kr/downloadwebQiaCom.do?id=44774 (2022). Accessed on February 12, 2024.

112. 소 항생제 처방 가이드라인 [Bovine antibiotic prescribing guidelines]. https://ebook.qia.go.kr/20210104_100444/ (2020). Accessed on February 12, 2024.

113. 돼지 항생제 처방 가이드라인 [Swine antibiotic prescribing guidelines]. https://ebook.qia.go.kr/20210104_100841/ (2020). Accessed on February 12, 2024.

114. Empfehlungen für den sachgemäßen einsatz von antibiotika in der tiergesundheit [Recommendations for the appropriate use of antibiotics in animal health]. https://agriculture.public.lu/dam-assets/veroeffentlichungen/broschueren/tiere/2022-einsatz-antibiotika-tiergesundheit.pdf (2022). Accessed on February 5, 2024.

115. Bierens JJM, van Beijnum LM, Dierikx CM, Palevliet JM, van Rossum nRJW, Vending JC: Werkgroep vterinair antibioticumbeleid - Formularium melkvee [The Veterinary Antimicrobial Policy Working Group - Dairy cattle formulary. https://www.knmvd.nl/app/uploads/sites/4/2023/06/230531-formularium-melkvee-versie-1.931.pdf (2023). Accessed on February 12, 2024.

116. Bakker J, van Duijkeren E, Ensink JM, Panhuijzen JJA, van Oldruitenborgh-Oosterbaan MMS, Stout TAE, et al.: Werkgroep vterinair antibioticumbeleid - Formularium paard [The Veterinary Antimicrobial Policy Working Group - Horse formulary]. https://www.knmvd.nl/app/uploads/sites/4/2021/03/wvab-formularium-paard-2016-versie-1.3.pdf (2021). Accessed on February 12, 2024.

117. D'Hoe K, Last WJ, Mölder P, Vendrig JC: Formularium vleeskalveren en vleesvee [Formulary for veal calves and beef cattle]. https://www.knmvd.nl/app/uploads/sites/4/2018/09/170315-wvab-formularium-vleeskalveren-en-vleesvee_definitief.docx.pdf (2019). Accessed on February 5, 2024.

118. van den Bosch MBW, Bouwman S, van den Brom R, Dijkstra E, van der Hidjen M, van den Oord P, et al.: Formularium kleine herkauwers - Schaap [Formulary of small ruminants - Sheep]. https://www.knmvd.nl/app/uploads/sites/4/2019/07/190619-formularium-schaap-definitief.pdf (2019). Accessed on February 5, 2024.

119. van den Bosch MBW, Bouwman S, van den Brom R, Dijkstra E, van der Hidjen M, van den Oord P, et al.: Werkgroep vterinair antibioticumbeleid - Formularium geit [The Veterinary Antimicrobial Policy Working Group - Goat hormulary]. https://www.knmvd.nl/app/uploads/sites/4/2020/09/formularium-geit-versie-1.1_220920.pdf (2019). Accessed on 5 February 2024.

120. van Duijkeren E, Hartog PA, van Hout AJ, Kanters MJF, van Nes A, Schyns MAR: Werkgroep vterinair antibioticumbeleid - Formularium varken [The Veterinary Antimicrobial Policy Working Group - Pig formulary]. https://www.knmvd.nl/app/uploads/sites/4/2019/09/formularium-varken_230919.pdf (2019). Accessed on 5 February 2024.

121. Richtlijn toepassen van antimicrobiële middelen [Guideline for the use of antimicrobials]. https://www.knmvd.nl/app/uploads/2022/03/150513-Richtlijn-TAM-definitief-versie-1.1.pdf (2015). Accessed on February 5, 2024.

122. Guide to prudent use of antimicrobial agents in pigs. https://www.amrvetcollective.com/assets/guidelines/AMR_Guide_Pigs.pdf (2020). Accessed on February 1, 2024.

123. Antibiotic judicious use guidelines for the New Zealand veterinary profession in dairy. https://www.amrvetcollective.com/assets/guidelines/guide_dairy.pdf (2018). Accessed on February 1, 2024.

124. Antibiotic judicious use guidelines for the New Zealand veterinary profession in equine. https://www.amrvetcollective.com/assets/guidelines/guide_equine.pdf (2018). Accessed on February 1, 2024.

125. Terapianbefaling: bruk av antibakterielle midler til produksjonsdyr [Therapeutic recommendation: use of antibacterial agents for production animals]. https://www.dmp.no/globalassets/documents/veterinarmedisin/terapianbefalinger/terapianbefaling---bruk-av-antibakterielle-midler-til-produksjonsdyr.pdf (2022). Accessed on February 1, 2024.

126. Wysocki M: Kodeks rozważnego stosowania produktów leczniczych przeciwdrobnoustrojowych przez lekarzy weterynarii [Code of the prudent use of antimicrobial medicinal products by veterinarians]. https://www.izbawetbial.pl/files/B202001.pdf (2020). Accessed on February 12 2024.

127. Ramalho FA: Utilização prudente de antimicrobianos em animais - vacas leiteiras [Prudent use of antimicrobials in animals dairy cows]. https://www.dgav.pt/wp-content/uploads/2023/07/HIPRA-UNIVERSITY_Filomena-Ramalho_MEDICAMENTOS-VETERINARIOS_.pdf (2023). Accessed on February 1 2024.

128. Guidelines for the use of antimicrobials in the South African pig industry. https://www.sava.co.za/wp-content/uploads/2017/05/Antibiotic-Guidelines-Pig-Industry-Ver-20161027-1.pdf (2016). Accessed on February 1, 2024.

129. Technical guidelines for responsible and prudent use of antimicrobials in veterinary medicine in South Africa. https://worldvet.org/uploads/docs/technical_guidelines_for_the_prudent_use_of_antimicrobials.pdf (2002). Accessed on February 1, 2024.

130. Recomendaciones para un uso prudente de los antibióticos en ganado bovino lechero [Recommendations for prudent use of antibiotics in dairy cattle]. https://neiker.eus/newsletters/documentos/guia-veterinario-es.pdf (2021). Accessed on February 5, 2024.

131. Guía de uso responsable de medicamentos veterinarios: ovino y caprino [Guide for the responsible use of veterinary medicines: sheep and goats]. https://www.vetresponsable.es/vetresponsable/guias-de-uso-responsable-por-especie-animal/ovino-y-caprino_3931_340_4077_0_1_in.html (2021). Accessed on February 1, 2024.

132. Guía de uso responsable de medicamentos veterinarios: bovino [Guide for the responsible use of veterinary medicines: bovine]. https://www.vetresponsable.es/vetresponsable/guias-de-uso-responsable-por-especie-animal/bovino_3930_340_4076_0_1_in.html (2017). Accessed on February 1, 2024.

133. Guía de uso responsable de medicamentos veterinarios: equinos [Guide for the responsible use of veterinary medicines: equines]. https://www.vetresponsable.es/vetresponsable/guias-de-uso-responsable-por-especie-animal/equino_3929_340_4075_0_1_in.html (2017). Accessed on February 1, 2024.

134. Guía de uso responsable de medicamentos veterinarios: porcino [Guide for the responsible use of veterinary medicines: porcine]. https://www.vetresponsable.es/vetresponsable/guias-de-uso-responsable-por-especie-animal/porcino_3928_340_4074_0_1_in.html (2017). Accessed on February 1, 2024.

135. Sveriges Veterinärförbunds riktlinjer för antibiotikaanvändning till nötkreatur & gris [Swedish Veterinary Association guidelines for antibiotic use in cattle & pigs]. https://www.svf.se/media/segp21ok/abriktlinjer-no-tkreatur-och-gris-rev2019.pdf#/media/media/edit/11574 (2019). Accessed on February 1, 2024.

136. Guidelines for the use of antibiotics in production animals - cattle, pigs, sheep and goats. https://www.svf.se/media/vd5ney4l/svfs-riktlinje-antibiotika-till-produktionsdjur-eng-2017.pdf (2017). Accessed on February 1, 2024.

137. The Swedish Veterinary Association’s guidelines for the clinical use of antibiotics in the treatment of horses. https://www.svf.se/media/tztkij4b/guidelines-antibiotics-in-horses.pdf (2013). Accessed on February 1, 2024.

138. Guide thérapeutique pour les vétérinaires - utilisation prudente des antibiotiques: bovins, porcs, petits ruminants et camélidés du Nouveau Monde [Therapeutic guide for veterinarians - prudent use of antibiotics: cattle, pigs, small ruminants and New World camelids]. https://www.blv.admin.ch/dam/blv/fr/dokumente/tiere/tierkrankheiten-und-arzneimittel/tierarzneimittel/therapieleitfaden.pdf.download.pdf/therapieleitfaden-fr.pdf (2022). Accessed on February 1, 2024.

139. Directives concernant l'emploi judicieux des médicaments vétérinaires [Guidelines for the judicious use of veterinary drugs]. https://www.gstsvs.ch/fileadmin/user_upload/GST-SVS/Publikationen/Richtlinien_Umgang_TAM_f.pdf (2022). Accessed on February 1, 2024.

140. BVA policy position on the responsible use of antimicrobials in food producing animals. https://www.bva.co.uk/media/1161/bva-policy-position-on-the-responsible-use-of-antimicrobials-in-food-producing-animals-1.pdf (2019). Accessed on February 1, 2024.

141. The judicious use of medically important antimicrobial drugs in food-producing animals. https://www.fda.gov/media/79140/download (2012). Accessed on February 1, 2024.

142. Cicchetti D. Guidelines, Criteria, and Rules of Thumb for Evaluating Normed and Standardized Assessment Instrument in Psychology. Psychological Assessment. 1994;6:284-90. doi: 10.1037/1040-3590.6.4.284.

143. Allerton F, Prior C, Bagcigil AF, Broens E, Callens B, Damborg P, et al. Overview and Evaluation of Existing Guidelines for Rational Antimicrobial Use in Small-Animal Veterinary Practice in Europe. Antibiotics. 2021;10(4):409.
